# Polyphasic Analysis of Intraspecific Diversity in *Epicoccum nigrum* Warrants Reclassification into Separate Species

**DOI:** 10.1371/journal.pone.0014828

**Published:** 2011-08-11

**Authors:** Léia Cecilia de Lima Fávaro, Fernando Lucas de Melo, Carlos Ivan Aguilar-Vildoso, Welington Luiz Araújo

**Affiliations:** 1 Empraba Agroenergia, Brazilian Agricultural Research Corporation, Brasília, Distrito Federal, Brazil; 2 Department of Cell Biology, University of Brasília, Brasília, Distrito Federal, Brazil; 3 Empraba Mandioca e Fruticultura, Brazilian Agricultural Research Corporation, Cruz das Almas, Bahia, Brazil; 4 Laboratory of Molecular Biology and Microbial Ecology, NIB, University of Mogi das Cruzes, Mogi das Cruzes, São Paulo, Brazil; Duke University Medical Center, United States of America

## Abstract

**Background:**

*Epicoccum nigrum* Link (syn. *E. purpurascens* Ehrenb. ex Schlecht) is a saprophytic ascomycete distributed worldwide which colonizes a myriad of substrates. This fungus has been known as a biological control agent for plant pathogens and produces a variety of secondary metabolites with important biological activities as well as biotechnological application. *E. nigrum* produces darkly pigmented muriform conidia on short conidiophores on sporodochia and is a genotypically and phenotypically highly variable species. Since different isolates identified as *E. nigrum* have been evaluated as biological control agents and used for biocompound production, it is highly desirable that this species name refers to only one lineage. However, according to morphological and genetic variation, *E. nigrum* present two genotypes that may comprise more than one species.

**Methodology/Principal Findings:**

We report the application of combined molecular (ITS and β-tubulin gene sequence analysis, PCR-RFLP and AFLP techniques), morphometric, physiological, genetic compatibility and recombination analysis to study the taxonomic relationships within an endophytic population that has been identified as *E. nigrum.* This combined analysis established two genotypes showing morphological, physiological and genetic divergence as well as genetic incompatibility characterized by colony inhibition, strongly indicating that these genotypes correspond to different species. Genotype 1 corresponds to *E. nigrum* while genotype 2 represents a new species, referred to in this study as *Epicoccum* sp.

**Conclusions/Significance:**

This research contributes to the knowledge of the *Epicoccum* genus and asserts that the classification of *E. nigrum* as a single variable species should be reassessed. In fact, based on the polyphasic approach we suggest the occurrence of cryptic species within *E. nigrum* and also that many of the sequences deposited as *E. nigrum* in GenBank and culture collection of microbial strains should be reclassified, including the reference strain CBS 161.73 sequenced in this work. In addition, this study provides valuable tools for differentiation of *Epicoccum* species.

## Introduction

*Epicoccum nigrum* Link (syn. *E. purpurascens* Ehrenb. ex Schlecht.) is an anamorphic ascomycete worldwide distributed which colonizes different types of soils and hosts plants. *E. nigrum* is mainly associated with the primary decomposition of plant tissues [1] and, although it has been described as a weak plant pathogen of some plants including *Cucumis melo* [2], this species is considered a saprotrophic fungus. Similar to other ubiquitous mould genera, this fungus can display an endophytic lifestyle and is commonly isolated from the inner tissues of several plant species [3], [4] including sugarcane [5], [6], one of the most important crops in Brazil because of biofuel production (http://english.unica.com.br).

In addition, *E. nigrum* has been used as a biological control agent against *Monilinia* spp. in peaches and nectarines [7], [8], [9], against *Sclerotinia sclerotiorum* in sunflowers [10] and against *Pythium* in cotton [11]. Many studies have focused on the ability of this fungus to produce antimicrobial compounds such as epicorazins A–B [12], epicoccins A–D [13], epicoccarines A–B and epipyridone [14], flavipin [15] and epirodins [16]. The production of novel bioactive chemical compounds including siderophores [17], antioxidants [18], inhibitors of HIV-1 replication [19], [20], inhibitors of leucemic cells [21], inhibitors of protease [22], inhibitors of telomerase [23] and fluorescent compounds with biotechnological applications [24], [25] have also been described in *Epicoccum* isolates.

The mitosporic fungus *E. nigrum* (Dothideomycetes) produces darkly pigmented multi-septate conidia (dyctiochlamidospores) on short conidiophores located on sporodochia [1]. In the *Epicoccum* genus, more than 70 species had been described but classification of these species was reduced to just one variable species, *E. nigrum* [26]. Currently, the species *E. nigrum* and *E. andropogonis* are accepted [27]. *E. nigrum* is known to be a highly variable species [28], [29] with distinct morphological and physiological types [30]. However, as reported for other cryptic and highly variable species of fungi, morphological differences often have been misunderstood or confused with intraspecific variation [31], suggesting that *E. nigrum* could comprises more than a single species.

Since different isolates identified as *E. nigrum* have been evaluated as biological control agents and used for biocompound production, it is highly desirable that this species name refers to only one lineage. In this study, we report on the application of combined molecular (ITS and β-tubulin gene sequence analysis, PCR-RFLP and AFLP techniques), morphometric, physiological and genetic compatibility approaches to study *E. nigrum* variability. This combined approach was used to explore the taxonomic interrelationships among endophytic isolates that has been identified as *E. nigrum.*

## Results

### Morphocultural characterization

**Figure 1:**
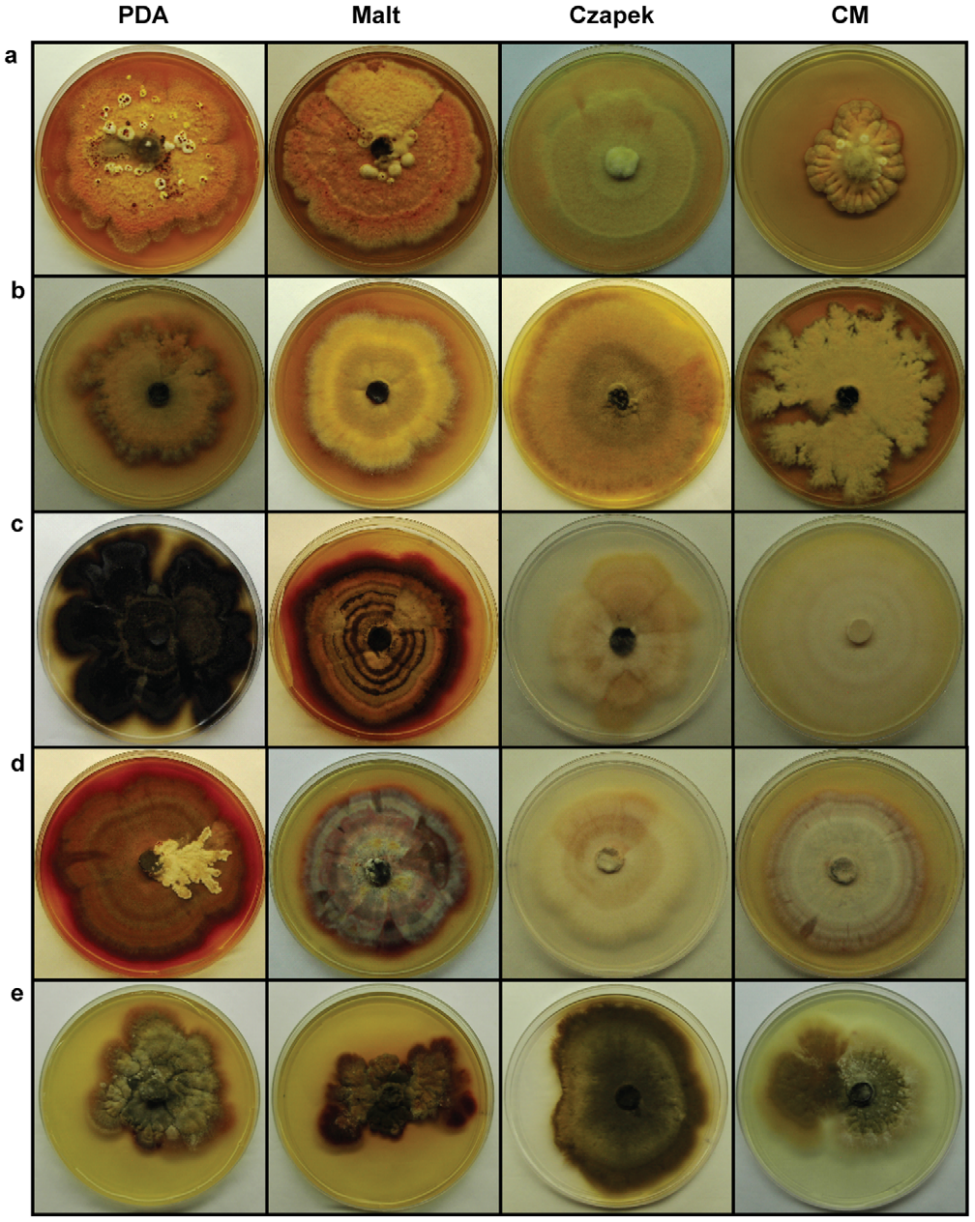
Colony aspect (top view) of some *Epicoccum* endophytic strains from sugarcane after growth in different culture media (PDA, Malt, Czapeck and Complete media).

Analysis of the *E. nigrum* sensu lato population (Table S1) showed that the evaluated strains belonged to two groups. Group 1 included colonies with typical *E. nigrum* morphology characterized by vigorous aerial mycelial growth, irregular margins, intense orange color (top view) and orange to dark red color (reverse) in PDA and malt media. These characteristics were also observed in complete medium, except that the top was yellow and the reverse side was brown. In Czapeck medium, the morphology was visibly altered with sparse mycelial growth immersed in the culture medium, uniform margins and color ranging from white to pale yellow (Table S2 and Figure 1). Group 2 showed dense mycelial growth in the culture medium, colonies with uniform edges and color ranging from purple, pink, red, gray and brown in PDA and malt media. In Czapeck and complete media mycelia grew thin in the culture medium with uniform borders and white to pale yellow mycelium (Table S2 and Figure 1).

**Figure 2:**
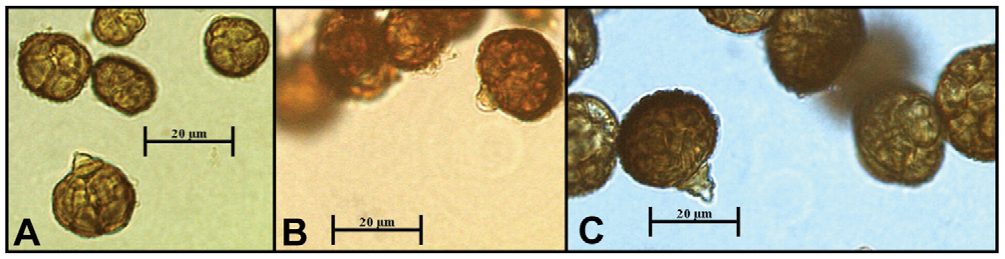
Conidia of *Epicoccum* .

The two populations could also be differentiated through analysis of the growth rates. In general, growth in all media evaluated produced a statistically higher growth index for the isolates of group 2 (0.7 to 1.44 cm per day) than for those belonging to group 1 (0.43 to 1.31 cm per day) (Table S2), with PDA medium producing statistically higher growth index than other media (Table S3). Group 1 exhibited a longer lag phase (10.2 to 31.4 h) than group 2 (2.14 to 19.2 h). Although morphological characteristics have separated isolates of *E. nigrum* into two major groups, we observed continuous variation and an overlap in regards to growth rates and lag phase duration within each morphological group, and also that some strains (e.g. Ep1sc, CBS 161.73) were unexpectedly slow growers compared with their groups (Table S2). All evaluated strains (Table S4) formed conidiophores on sporodochia, which began to develop after approximately 25 days on PDA. Strains belonging to group 1 and group 2 had similar conidial morphologies (Figure 2), although conidia from group 1 were, in general, bigger than from group 2. There was also overlap between groups, with group 1 ranged from 25.31 to 38.66 µm in length and 19.16 to 29.01 µm in width, whereas group 2 isolates ranged from 17.67 to 26.69 µm in length and 12.81 to 20.13 µm in width (Table S4).

### Enzymatic analysis

**Figure 3:**
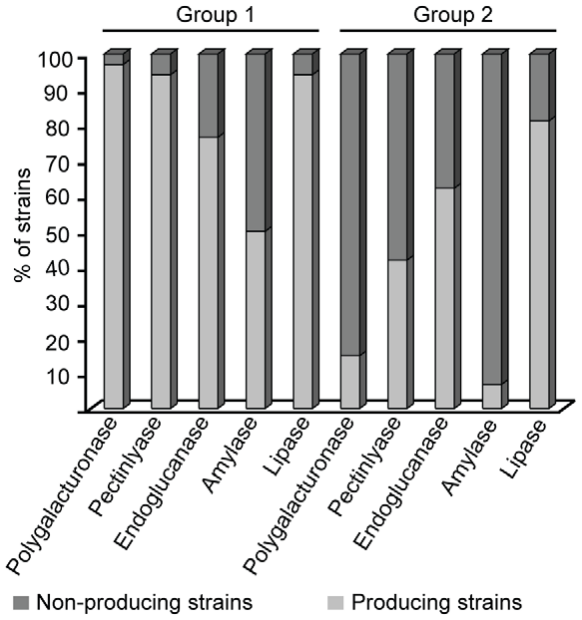
Enzymatic profile of *Epicoccum* strains from groups 1 and 2.

Physiological characterization revealed significant differences between groups 1 and 2. Strains belonged to group 1 have a greater ability to secrete hydrolytic enzymes in solid media (Figure 3) than group 2 strains (Table S5). Protease (gelatinase) activity was not detected in the evaluated strains, while amylase activity was not observed in strains belonging to group 2. Furthermore, lipase was secreted by 87.5% of the investigated isolates. Significant differences were detected in pectinase production (Figure 3); the strains from group 1 were more able to secrete enzymes necessary for a saprophytic lifestyle than the strains from group 2 strains. Also, we observed that strains with slower growth rates had higher enzymatic activity levels (see Tables S2 and S5).

### Isolation and characterization of Nit mutants

Nitrate non-utilizing mutants were selected by resistance to chlorate. For this, 110 *Epicoccum* strains were tested and 78 strains spontaneously generated at least one sector resistant to chlorate when mycelial plugs were incubated on medium amended with chlorate (Table S6). The number of mutants resistant to chlorate varied among the strains (ranging from 1 to 12 sectors per strain), suggesting the occurrence of instability in some strains or that instability generating mechanisms had been activated during the selection of these mutants in chlorate. A total of 271 chlorate-resistant sectors were obtained, from which 57 (from 35 different strains) were characterized by growth tests in different nitrogen sources (Table S6). Most of these mutants (56.14%) could not use nitrate as a nitrogen source (Nit mutants) and grew as thin and sparse colonies on culture media containing nitrate when compared with the dense growth of the respective wild-type strain.

The Nit mutants could be divided into four phenotypic classes that probably represented mutations in the nitrate reductase structural locus (*nit*1), in the pathway-specific regulatory locus (*nit*3), in loci that codify the cofactor containing molybdenum (NitM), and one or more genes responsible for nitrate intake (*crn*). The *crn* mutants were predominant, followed by *nit*1, *nit*3, and NitM (Table S6). *nit*1, *nit*3, and NitM mutants from 22 different strains (14 strains from group 1 and 8 strains from group 2) were selected for further complementation tests.

### Mycelial reactions

**Figure 4:**
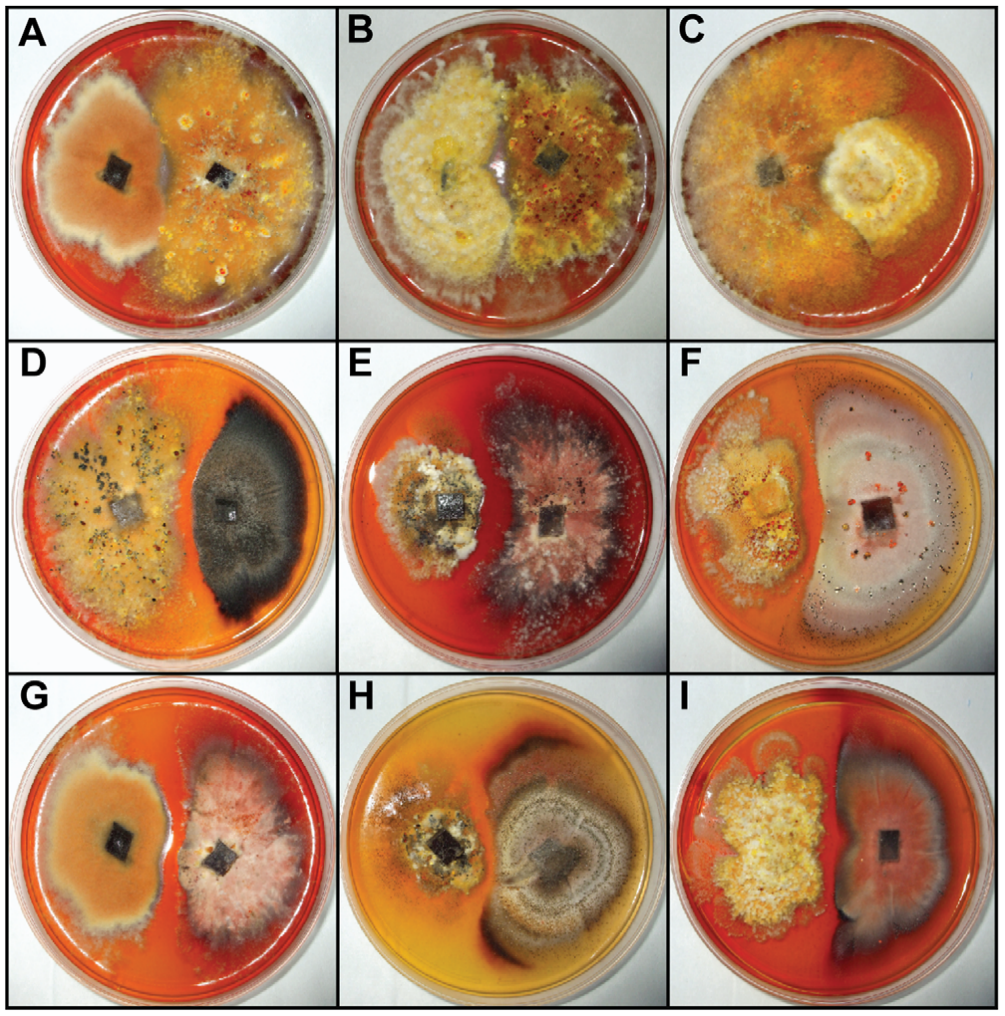
Examples of mycelial reactions among *Epicoccum* strains.

Strains from each morphological group (n = 50; 25 from each group) were paired with one another and through mycelial interaction tests the defined groups were found to be incompatible. The pairings exhibited two types of mycelial reactions: (1) formation of colonies in close contact to the line of interaction between strains, but without hyphal anastomosis, and (2) the formation of a zone characterized by the antagonism of mycelial growth and inhibition of the strains. The first type of mycelial reaction was observed when strains from the same group were paired; however, when strains from groups 1 and 2 were paired, an antagonistic area was observed (Figure 4).

We also analyzed the mycelial interactions among strains by using nitrate non-utilizing mutants (Nit). We did not observe complementation or heterokaryotic zone between Nit mutants generated from different strains. In fact, the pairings between wild-type strains or between Nit mutants resulted in similar mycelial reaction profile, in other words, when Nit mutants from the same group were paired, the colonies grown in close contact to the line of interaction, but without hyphal fusion; in contrast, when Nit mutants from different groups were paired, an antagonistic area was observed between mutants. We only observed complementation in the pairings between two *nit*1 mutants originating from a same strain (C41B), suggesting that *nit*1 mutants are allelic in *Epicoccum*. This analysis demonstrates that the lack of compatibility between strains of groups 1 and 2 may be a result of reproductive isolation. This accounts for increased genetic divergence between these groups.

### Phylogenetic analysis

**Figure 5:**
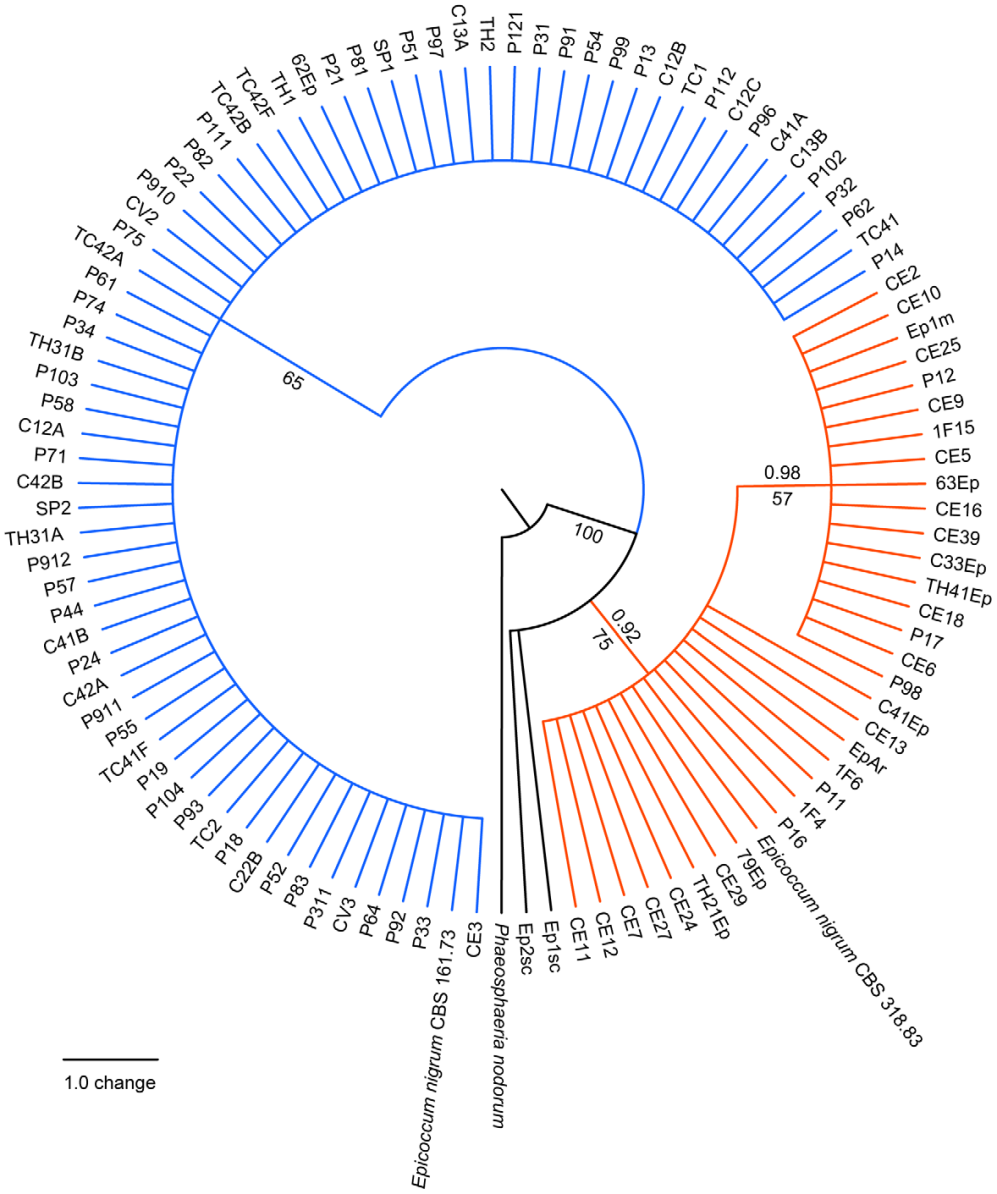
Strict consensus tree of the most parsimonious trees inferred using ITS1-5.8S-ITS2 sequences of 106 *Epicoccum* strains. Two *E. nigrum* reference strains (CBS 318.83 and CBS 161.73) were included and *P. nodorum* access AF250830 was used as outgroup. The bootstrap and the posterior probability values are shown next to relevant nodes. Orange lines represent the *Epicoccum* strains from group 1. Blue and black lines represent the strains from group 2.

The genetic relationships between endophytic isolates of *E. nigrum* from sugarcane and other plant hosts were examined using sequences of the ITS region of rDNA and the β-tubulin gene with maximum parsimony and Bayesian analysis. Amplification of the ITS1-5.8S-ITS2 region with primers ITS1 and ITS4 yielded a fragment of approximately 570 bp for the 106 strains studied. Phylogenetic analysis based on this region was performed using 456 bp (complete sequence), with the ITS sequence of *Phaeosphaeria nodorum* as outgroup (Figure 5 and Figure S1). The phylogenetic analysis of *Epicoccum* clearly distinguished the isolates into two well supported groups (Figure 5 and Figure S1), similar to results from morphocultural analysis. The first clade (group 1), which has a smaller number of taxa than the other, included the reference strain CBS 318.83, while the second clade (group 2) included the reference strain CBS 161.73. In general, no clustering by geographic origin or substrate/host was observed in the trees. The overall genetic distance between 106 samples of ITS1-5.8S-ITS2 sequences used in this analysis was 0.007 (SE = 0.002). When genetic distance was calculated based on the clustering obtained in the phylogenetic analysis, the divergence within group 1 was 0.003 (se = 0.002) and within group 2 was 0.000 (SE = 0.000). However, the genetic distance between the two groups was 0.015 (SE = 0.005), indicating that these clades may represent distinct lineages.

An additional phylogenetic analysis was performed with the complete sequences obtained in the present study and ITS sequences from GenBank, including sequences from *Epicoccum* as well as from different *Phoma* species, which produce dyctiochlamidospores and have been phylogenetically related to *P. epicoccina* ( = *E. nigrum*), as reported previously [32]. The results showed two clades (Figure S2), which were similar to clades observed with only *Epicoccum* strains obtained in the present study. In this complete analysis, the reference strain CBS 318.83 grouped in the well supported clade 1 together with ATCC 96794, ATCC 32948 and ATCC 62191 reference strains accessions, while clade 2 included the reference strain CBS 161.73 and accessions identified as *P. epicoccina*, *E. andropogonis*, *E. nigrum* and *Epicoccum* sp.

**Figure 6:**
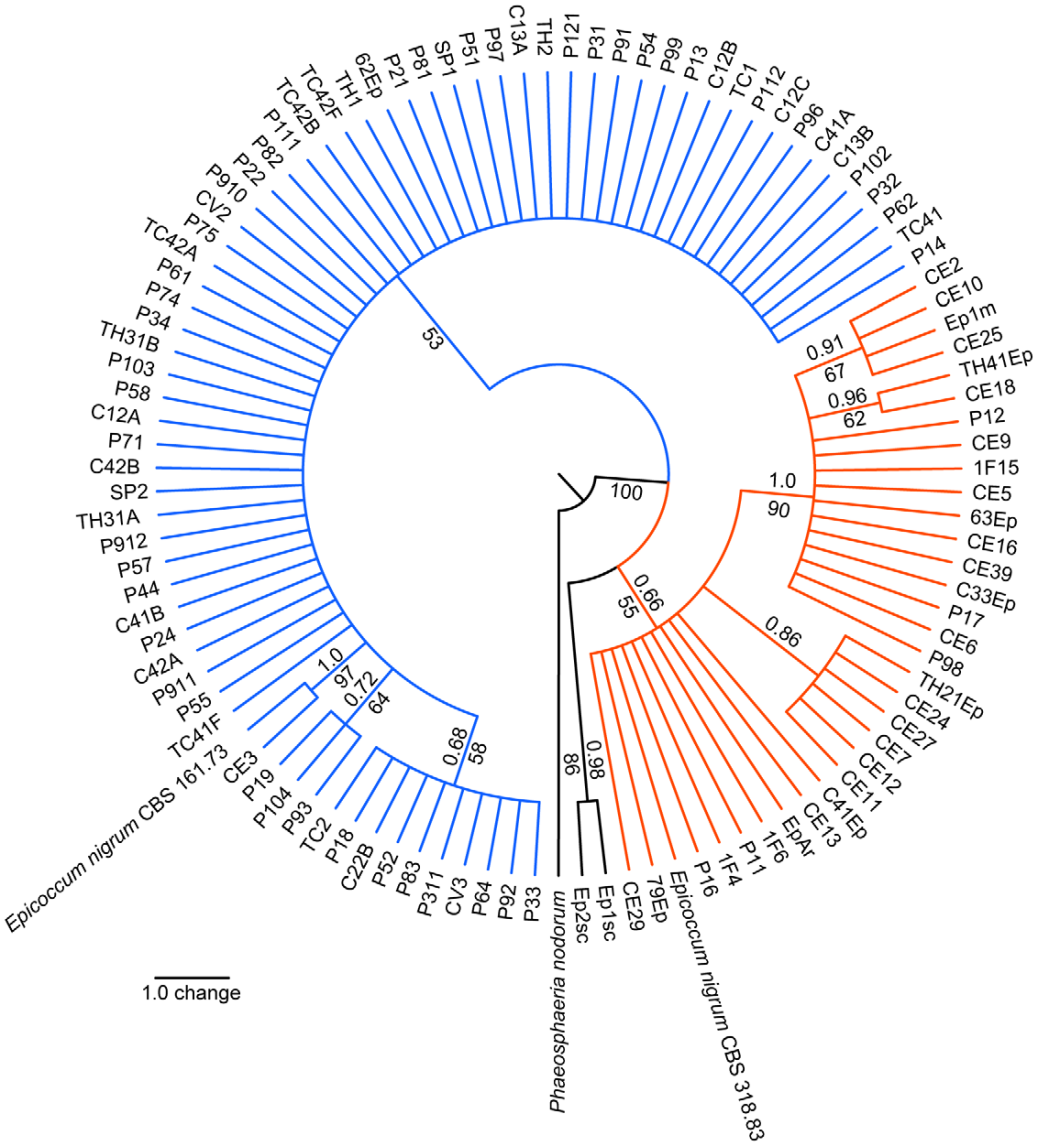
Strict consensus tree of the most parsimonious trees inferred using β-tubulin sequences of 106 *Epicoccum* strains. Two *E. nigrum* reference strains (CBS 318.83 and CBS 161.73) were included and *P. nodorum* access AY786336 was used as outgroup. The bootstrap and the posterior probability values are shown next to relevant nodes. Orange lines represent the *Epicoccum* strains from group 1. Blue and black lines represent the strains from group 2.

**Figure 7:**
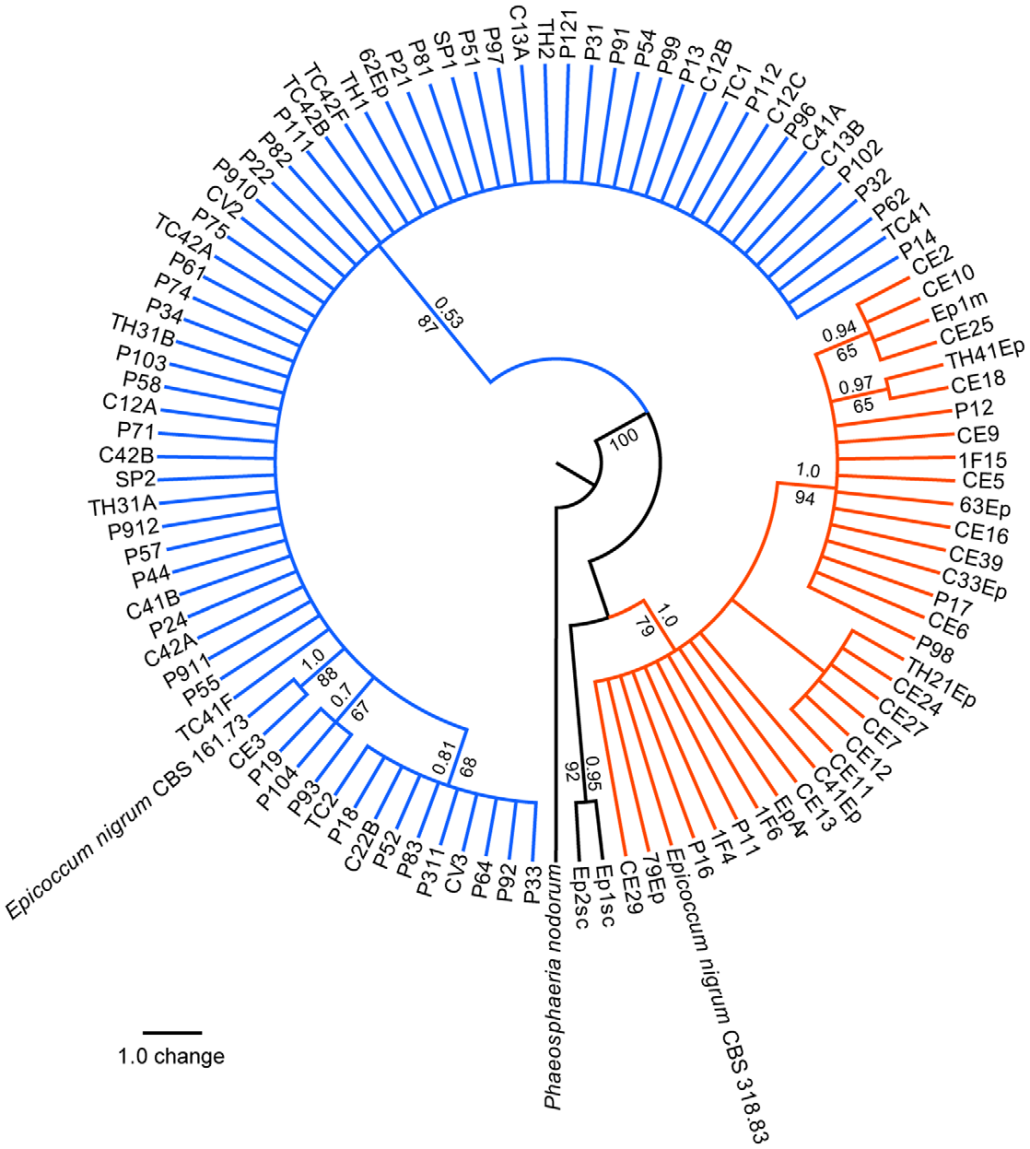
Strict consensus tree of the most parsimonious trees inferred using concatenated ITS1-5.8S-ITS2 and β-tubulin sequences of 106 Epicoccum strains. Two *E. nigrum* reference strains (CBS 318.83 and CBS 161.73) were included and *P. nodorum* accessions AF250830 and AY786336 were used as outgroup. The bootstrap and the posterior probability values are shown next to relevant nodes. Orange lines represent the *Epicoccum* strains from group 1. Blue and black lines represent the strains from group 2.

A 350-bp fragment of the β-tubulin gene was obtained from 106 isolates of *Epicoccum*, including the *E. nigrum* reference strains CBS 318.83 and CBS 161.73. Phylogenetic analysis were performed using 344 bp, with the β-tubulin sequence of *P. nodorum* as outgroup (Figure 6 and Figure S3). The result supported two clades (Figure 6 and Figure S3) similar those observed with ITS, morphocultural and physiological analysis. As conflict was not observed by partition homogeneity test among ITS and β-tubulin sequences, an additional combined phylogenetic analysis was performed using *P. nodorum* as outgroup (Figure 7 and Figure S4). This analysis also generated two well-supported clades (Figure 7 and Figure S4), similar to those previously observed.

As β-tubulin sequences from *Epicoccum* species is not available in GenBank, we also carried out another analysis including β-tubulin sequences from different species of *Phoma* able to produce dyctiochlamidospores. Similar to previous analysis, the results showed two groups (Figure S5), being the group 2 close to *P. epicoccina* (Syn.: *E. nigrum*).

### AFLP analysis

The AFLP analysis was performed to assess the genetic variability and relationships between endophytic isolates of *E. nigrum* from sugarcane and other host plants. All 322 bands obtained with the combination of EcoRI+A/MseI+CA or EcoRI+A/MseI+AT primers were polymorphic. The UPGMA cluster analysis based on Dice's similarity coefficient (r = 0.99662) separated the isolates into two major clades (Figure S6), corresponding to groups 1 and 2 previously obtained by sequence, morphocultural and physiological analyses. The distinction of these clades was supported by high bootstrap values (100 and 87.3%, respectively). Group 1 (*E. nigrum*) consisted of isolates with similarity coefficients ranging from 0.48 to 1.0 and group 2 isolates had similarity coefficients ranging from 0.18 to 1.0. All strains in group 1 and 92% of strains in group 2 had a similarity coefficient around 0.48 to 0.50, indicating high intragroup variability among the studied strains. The index of similarity between the two clades was very low (0.13), indicating that two distinct groups were present within the population. From the 55 evaluated strains, 30 belong to group 1 and 25 belong to group 2 (with 25 and 21 haplotypes, respectively). However, this AFLP analysis generated subgroups (1A, 1B, 2A, 2B and 2C) with a similarity around 50% and high support values in both groups. Although strains Ep1sc and Ep2sc (subgroup 2C) have been classified as group 2 based on the morphology, in the AFLP analysis they were also clustered in group 2, but with a very low similarity to the other strains from this group, possibly reflecting their more distant geographic origin (Table S1).

**Figure 8:**
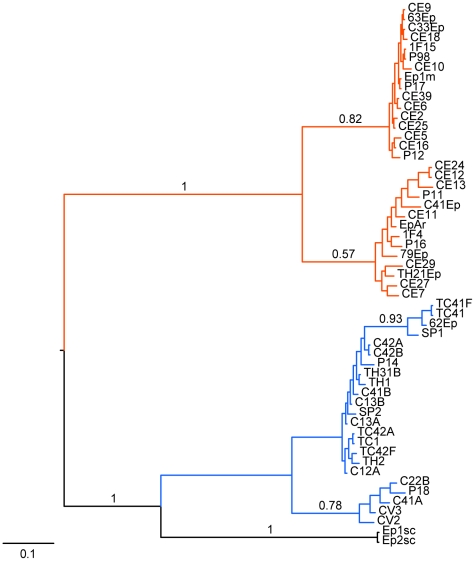
Bayesian maximum clade credibility tree inferred using AFLP data from 55 *Epicoccum* endophytic strains. The tree is mid-point rooted for purposes of clarity. The posterior probability values are shown next to relevant nodes. Orange lines represent the *Epicoccum* strains from group 1. Blue and black lines represent the strains from group 2.

The differentiation level between groups 1 and 2 was estimated by AMOVA, which detected significant variation (P>0.001) between the two groups, producing an *F*_ST_ value of 0.60651 indicating a high level of differentiation between these clades. Moreover, this analysis revealed that most of the detected variation (60.65%) occurs between groups and 39.35% corresponds to intragroup variation (Table 1). The Bayesian analysis (Figure 8) generated a tree topology congruent with the tree generated by the phenetic analysis (Figure S6). Specifically, two major clades corresponding to groups 1 and 2 were formed. These clades were well supported with a posterior probability value of 1.0. We also observed the formation of well supported subgroups within each clade (Figure 8), similar to the findings of the phenetic analysis.

**Table 1:** Analysis of molecular variance of *Epicoccum* strains from two different groups using 322 AFLP markers.

Source of variation	Degrees of freedom	Sums of squared deviation	Variance components	Percentage of variation	*F*_st_
Among groups	1	1022.247	36.61148	60.65	0.60651*
Within groups	53	1258.880	23.75245	39.35	
Total	54	2281.127	60.36393		

P>0.001.

### PCR-RFLP analysis

The amplification of the ITS1-5.8-ITS2 rDNA produced a fragment of approximately 570 bp for 56 isolates and *E. nigrum* reference strains CBS 318.83 and CBS 161.73. Out of 20 tested endonucleases, eight (HhaI, RsaI, ClaI, Hin6I, SphI, MspI, EcoRI, MboI) cut at least once in the rDNA region. However, only MspI was able to differentiate the strains into the two major clades by generating 2 distinct haplotypes (not shown). The groups showed a low similarity value (0.40) and high homogeneity among the isolates within each group. In general, this analysis corresponded to the clades obtained by AFLP and sequence analysis of the ITS region and the β-tubulin gene.

**Figure 9:**
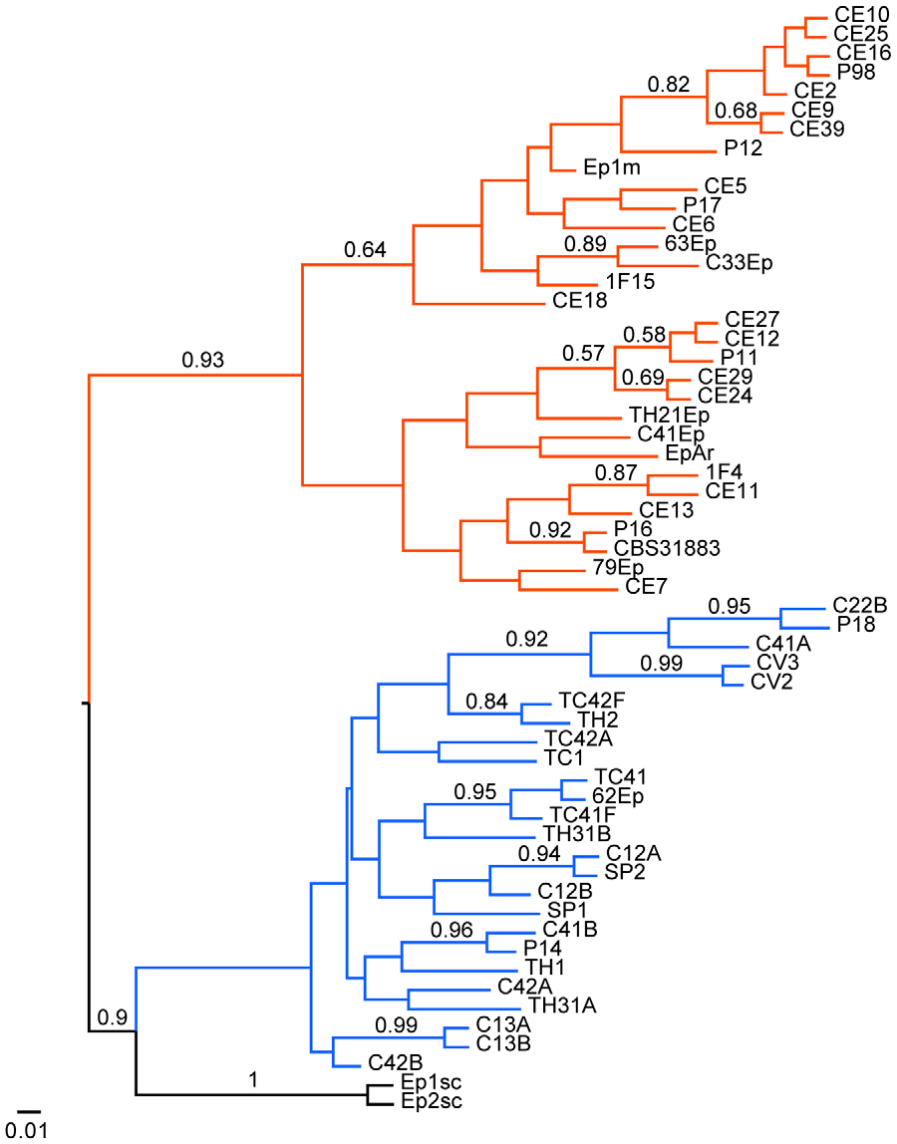
Bayesian maximum clade credibility tree inferred using IGS-RFLP data from 58 *E. nigrum* endophytic strains. The tree is mid-point rooted for purposes of clarity. The posterior probability values are shown next to relevant nodes. Orange lines represent the *Epicoccum* strains from group 1. Blue and black lines represent the strains from group 2.

We also investigated the application of the IGS-RFLP technique for the differentiation of *Epicoccum* isolates (Figure S7), which has not been previously described. Except for CBS 161.73, which generated two bands, a single fragment was produced from 58 strains using CNS1 and CNL12 primers. These fragments ranged from 2500 to 3000 bp. Because of this difference, CBS 161.73 was not included in the IGS-RFLP analysis. Out of 17 tested endonucleases, five (BsuRI, HinfI, PstI, MspI, Hin6I) cut the IGS region, but only three (BsuRI, HinfI, PstI) generated reliable patterns. Therefore, we used these enzymes for analysis resulting in the discrimination of the tested strains in two groups. The digestion patterns distinguished 48 unique haplotypes among 58 evaluated strains. In phenetic (Figure S8) and phylogenetic analysis (Figure 9) the two major clades obtained were statistically supported. Clades 1 and 2 included 31 and 27 strains with 26 and 22 haplotypes, respectively. Notably, no haplotype was shared between the clades. The IGS-RFLP analysis distinguished the strains and produced a tree with topology similar to that observed by other analysis.

### Recombination analysis

The indexes of association (I_A_ and r_d_) calculated from the AFLP data significantly deviated from the null hypothesis (population is randomly recombining). This result was observed when the isolates in each dataset were considered as a single population. Similar result was observed when analysis was done only with haplotypic data (Table 2), suggesting the absence of gene flow between strains. Also, the well-supported groups and subgroups revealed by the AFLP phenetic analysis, with similarity index values in a range of 0.5 to 0.65 (Figure S6) were used as populations in an additional recombination analysis, and the results showed (P<0.001) that recombination is not occurring between AFLP group 1 and AFLP group 2 (Table 2). Although recombination was not significantly detected between subpopulations inside AFLP group 1, the observed values of the association indexes were not placed so distant from the extremes of the variation of the artificially randomized datasets (Table 2). However, inside AFLP group 2, the analysis showed that recombination is occurring between subgroups (P = 0.08), indicating that recombination may play a role in determining the population structure of *Epicoccum*.

**Table 2:** Linkage disequilibrium analysis using different datasets of AFLP markers of endophytic *Epicoccum* strains.

				Haplotypic data	
Data sets	N° of strains	N° of haplotypes	Populations in the analysis^a^	IA	rd
G1+G2	55	46	1	34.2522 (P<0.001)	0.116629 (P<0.001)
			2 (G1; G2)	34.2522 (P<0.001)	0.116629 (P<0.001)
G1	30	25	1	33.8834 (P<0.001)	0.260601 (P<0.001)
			2 (1A; 1B)	33.8834 (P<0.001)	0.260601 (P<0.001)
			3 (1A; 1B*)	33.8834 (P<0.001)	0.260601 (P<0.001)
G2	25	21	1	26.5615 (P<0.001)	0.159194 (P<0.001)
			3 (2A; 2B; 2C)	26.5615 (P<0.001)	0.159194 (P<0.001)
			4 (2A*; 2B; 2C)	26.5615 (P = 0.055)	0.159194 (P = 0.055)
G2 without Ep1sc/Ep2sc	23	20	1	24.7395 (P<0.001)	0.190673 (P<0.001)
			2 (2A; 2B)	24.7395 (P<0.001)	0.190673 (P<0.001)
			3 (2A*; 2B)	24.7395 (P = 0.082)	0.190673 (P = 0.082)

a The number of populations in the analysis was determined based on the well-supported groups and subgroups obtained in the phenetic analysis of t he AFLP data (G1 and G2; subgroups 1A and 1B; subgroups 2A, 2B, and 2C). (*) The subgroup 2A was subdivided into two other well-supported subgroups according to the phenetic analysis of AFLP data. The index of multilocus association (I_A_) and the index of association (r_d_) were calculated by the MultiLocus software (see reference 90).

### Phylogenetic analysis of the combined data of DNA fingerprints and DNA sequences

**Figure 10:**
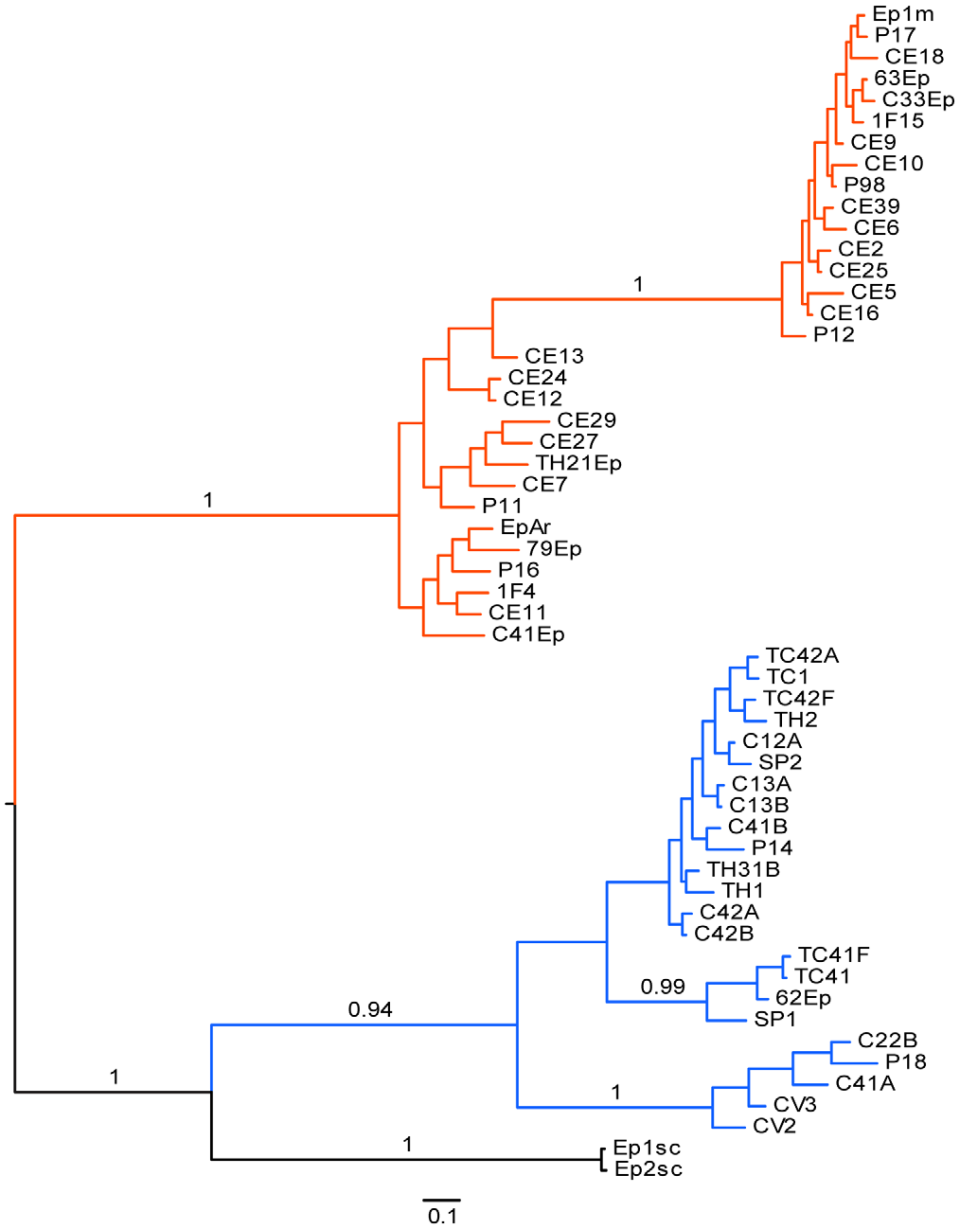
Bayesian maximum clade credibility tree inferred using DNA fingerprint (AFLP and IGS-RFLP) and sequences (ITS1-5.8S-ITS2 and β-tubulin) data from 55 *Epicoccum* endophytic strains. The tree is mid-point rooted for purposes of clarity. The posterior probability values are shown next to relevant nodes. Orange lines represent the *Epicoccum* strains from group 1. Blue and black lines represent the strains from group 2.

The combined analysis resulted in a tree with a topology very similar to our previous analysis. Better resolution of the internal branches in the two groups and a more refined separation of the strains were observed (Figure 10).

## Discussion

Traditionally, morphology and reproductive biology analysis provide the basis for the identification and classification of fungal species, but phylogenetic approaches have recently become popular for the recognition of species [33], . Especially in fungi for which few diagnostic characters are described, an accurate species identification may play a important role for assessment of biodiversity and conservation planning, bioprospecting, and implementation of biological control and protective measures against pathogens [36], [37]. The correct identification of biocontrol agents can be necessary for their release and to satisfy quarantine authorities. Moreover, natural population analysis of the biocontrol fungi is important for the effectiveness of the biocontrol programs, for example, to evaluate the stability of a strain in the environment. Recombination of a biocontrol agent could have impact on its genetic stability, thus hindering its application and monitoring. In other cases, if recombination is rare, the use of traditional crossing techniques for strain improvement may not be successful. In this context, we applied a polyphasic approach, including morphocultural, physiological, mycelial interactions, recombination tests and extensive molecular characterization, to assess the taxonomic relationships among different isolates of *E. nigrum.*

The results of morphocultural characterization clearly support two distinct groups: group 1 with typical morphology of *E. nigrum* (yellow to orange mycelium) and group 2 with variable morphology (gray, pink, purple, red or brown mycelium). Although the dimensions of conidia are within the variation described for *E. nigrum* [1], strains from group 1 had larger dimensions than those belonging to group 2. The growth rate, lag phase timing and enzyme production presented continuous variation but also distinguished strains of group 1 from those belonging to group 2. Actually, group 1 isolates produce high levels of a variety of degradative enzymes, mainly lipases, pectinases and endoglucanase. Some isolates (e.g. Ep1sc and CBS 161.73) exhibited slower growth compared with their groups. These strains were originated from a distant geographic area and showed low genetic similarity (Ep1sc strain) with group 2 strains, as revealed by the AFLP analysis.

The association between plants and endophytic fungi is ecologically and agriculturally important, although little is known about the physiological aspects of these interactions [38]. Furthermore, proteins secreted by fungi are probably important components of the mutualistic interaction because they are located at the interface of the two species [38]. In this context, our results suggest that the production of hydrolytic enzymes by endophytic isolates of *Epicoccum* may also be important for fungal nutrition during the endophytic-host plant interaction. If these enzymes are also secreted on the leaf surface, they may be involved, among other factors, in the competition for substrate on phyllosphere and niche exclusion [39]. Overall, our physiological data suggest that these groups may occupy different ecological niches, with group 1 presenting higher saprophytic competence than group 2. If this is true, different genotypes can coexist in the host tissues and exploit different substrates. This seems to be the case, since some *Epicoccum* strains with different morphotypes were isolated from sugarcane leaf tissues obtained from the same plant.

A better understanding of the lifestyle of the endophytes has been achieved through studies of enzyme production. For instance, based on the ability of endophytic fungi to produce the same degrading enzymes that are secreted by saprobes, it has been suggested that endophytes can become saprobes following senescence of the host tissue, thus playing key roles in fungal succession and plant decomposition [40]. It has also been demonstrated that endophytes cause plants to enter a “primed state” [4], [41], [42], which is characterized by an increased capacity to express basal defense responses following biotic challenges [41]. In this aspect, the hydrolytic enzymes produced by endophytes, such as those secreted by *Epicoccum*, among other factors, may play an important role in generating defense responses in plants. Further studies on the endophytism of *Epicoccum* should clarify the physiological responses of the host plant to this common inhabitant of the phyllosphere.

We further investigated if these morphocultural and physiological differences are supported at the genetic level by mycelial interactions between isolates. We observed that mycelial interaction between mutants and wild-type strains from group 1 and group 2 resulted in a clear antagonistic zone. Although analysis of the mycelial interactions among wild-type isolates have been used in vegetative compatibility studies [43], as has been done in this work, this approach may not to be clear enough to define incompatibility [43]. The limitation associated with the pairing of wild-type strains is that in many species the morphological manifestation of incompatibility, such as apoptotic barrages, is variable [43], in part due to the strong influence of the mating type in the interaction zone. However, the results with Nit (nitrate non-utilizing) mutants revealed similar results, suggesting that non-self-anastomosis is restricted, as has been described in other mitosporic Dothideomycete, *Alternaria brassicicola* [44]. Complementation was only observed between *nit*1 mutants originating from a same strain (self-anastomosis), which suggests that *nit*1 mutants in *Epicoccum* could be allelic. Although we are aware of the fact that a greater number of Nit mutants should be analyzed in vegetative compatibility studies, these initial results nevertheless indicate that there is no gene flow between the two groups, which may allow for genetic divergence and consequently speciation.

The heterokaryon formation between strains indicates vegetative compatibility, which may result in meiotic or mitotic recombination [45]. In mitosporic fungi, such as *Epicoccum*, several mechanisms, such as parasexuality, aneuploidy, migration, and mutations caused by the action of transposable elements, could be responsible for the genetic variation observed within populations [45], [46], [47], [48], [49]. Also, it has been suggested that mating-type genes may regulate the genetic exchange in some mitosporic fungi [50], such as *Alternaria alternata* [51] and *Trichoderma reesei* [52]. Although the presence of the mating-type genes in *Epicoccum* still needs to be investigated, their putative functionality, together with the above mentioned mechanisms, may contribute to the generation of the genetic diversity within *E. nigrum*.

In fact, *E. nigrum* is known as a genotypically and phenotypically highly variable species [28], [29], [30]. This heterogeneity has been explained by presence of heterokaryotic strains [30], which occur due to the anastomosis between germ tubes of recently germinated multinucleate conidia [53] and is observed during fungi growth by the frequent occurrence of sectorizations [26], [30]. Since we did not observe heterokaryon formation between Nit mutants from different strains, the anastomosis among germinated conidia could be one of the several possible mechanisms accounting for the high genetic diversity in this mitosporic fungus. According previous studies, this high variability could be important for survival under adverse ecological conditions [30]. Although previous studies on the morphological variability of *E. nigrum* have reported some evidence for the occurrence of morphotypes with yellow to orange mycelium and morphotypes without this feature occurring in a same local [30], these characteristics were misunderstood as intraspecific variation. Our results on mycelial interactions clearly demonstrate that these morphotypes are genetically incompatible.

The genetic relationships between endophytic isolates of *E. nigrum* from sugarcane and other plant hosts were examined using sequences of the ITS region of rDNA and the β-tubulin gene through maximum parsimony and Bayesian inference. The phylogenetic analysis showed that *E. nigrum* could be also clustered in two well-supported groups: group 1 was similar to strains with the *E. nigrum* typical morphology (yellow to orange mycelium) and group 2, which present variable morphology (gray, pink, purple, red or brown mycelium), was close to strains classified as *E. andropogonis* and *P. epicoccina* [54], [55]. An important observation was that, despite of the fact that our sample was composed mainly by strains isolated from a single tropical region and host plant, these strains were clustered with *Epicoccum* ITS accessions from a wide range of substrates and geographic regions (although most of them come from temperate regions), which suggest that the two groups are widely distributed. However, the discrete clustering including most of our strains (Figure S2) can also indicates that *E. nigrum* may consists of independent lineages with discrete distribution patterns, as have been shown in other fungi [56], [57].

The overall pairwise genetic distance calculated from the ITS sequences in this study was 0.7% divergence, but the genetic distance (1.5%) between the two groups indicated that groups have distinct lineages. The variation observed between groups 1 and 2 corresponds approximately to the values found among few isolates of *E. nigrum* and *P. epicoccina* in a previous study in which this variation was considered within the range of intraspecific variation reported for other fungal species [54]. Based on ITS comparisons, *Cerebella andropogonis* has been related to *Epicoccum* [55] indicating that these genera are synonyms, as proposed previously [26]. Based on morphological observations and ITS analysis, it was proposed that *P. epicoccina* and *E. nigrum* are the same biological species [54], [58], in part due to the fact that *P. epicoccina* produces dyctiochlamidospores that are indistinguishable from those produced by *E. nigrum* [58], but *E. nigrum* isolates have probably lost the ability to produce pycnidia, which are found in *P. epicoccina*. In fact, in the evaluated strains, pycnidia-like structures were not observed, indicating that these are not *P. epicoccina* strains. Therefore, our results confirm that the isolates previously identified as *E. nigrum* could be separated in two closely related species (Figure S2) with closest relatives in the *Phoma* section *Peyronellaea* [32] within the recently proposed family *Didymellaceae* [59]. Other important observation from the ITS analysis was that the well-supported clade 1 (orange morphotype) is phylogenetically derived from an ancestor related to the clade 2 (non-orange morphotype) (Figure S2). Moreover, our findings raise doubts about the synonymization of *P. epicoccina* with *E. nigrum*, as proposed previously [54], [58].

As previously reported [60], the ITS region is often not different enough to separate closely related species, and other protein-coding genes must be analyzed to better assess the genetic relationship between fungal species. In light of this, the present study examines for the first time the genetic relationship between *Epicoccum* strains based on the sequence of the β-tubulin gene. This analysis distinguished two clades, as observed by ITS, morphocultural and physiological analyses, supporting the hypothesis that *E. nigrum* should be separated into two species.

Using a genome-wide sampling technique such as AFLP, we observed similar clustering obtained with gene sequence, morphocultural and physiological analyses. The *F*_ST_ was estimated to be 0.60651. Based on previous studies of the genetic structure of fungal populations, *F*_ST_ values ranging from 0 to 0.2 are considered low to moderate levels of genetic differentiation between populations [48], . In combination with the compatibility data presented here, the high value of genetic differentiation between groups 1 and 2 suggests that these two populations are structured and that these clades are distinct taxa with no gene flow. These findings were reinforced by the analysis of the linkage disequilibrium, which revealed no evidence for recombination between the two groups. Since it is known that mechanisms other than clonality may explain the association of alleles, for example, the inclusion of isolates from genetically isolated groups in the recombination analysis [62], [63], we also performed this analysis using data from the two groups separately. There was also no evidence for recombination within groups when the isolates from each group were considered as a single population. However, when the analysis was performed considering the well supported subgroups within each AFLP group as distinct populations, recombination was detected, suggesting that this mechanism may contributes to the population structure of *Epicoccum*.

Considering that *E. nigrum* is a mitosporic fungus, the variation observed within groups was relatively significant (∼40%). This suggests that mechanisms such as mutation, migration or parasexuality could contribute to the generation of genetic diversity. Although we did not observe heterokaryon formation between Nit mutants from different strains within each group, a prerequisite for parasexuality, the linkage disequilibrium analysis showed that recombination may occurs between isolates within each AFLP subgroup sharing genetic similarity index greater than 50%, and account for the genetic variability in natural populations of *Epicoccum*. Furthermore, the use of molecular tools in fungal taxonomy studies have demonstrated that many recognizably asexual species have relatives in sexual taxa [62], [63], which suggests an increasing speciation rate in these mitosporic fungi or, the occurrence of adaptive radiation to new ecological conditions after loss of ability to sexual reproduction [63]. In the present work, we found evidence for niche separation (distinct substrate utilization), probably due to ecological specialization, and also reproductive isolation (represented by the absence of recombination and incompatibility between the two groups). Since the two identified groups may represent global species, as revealed by the ITS phylogeny, these findings represent the initial steps toward a better understanding of the forces driving speciation in the highly diverse *Epicoccum* group.

In general, AFLP analysis exhibited more variation than the ITS sequence analysis. This is exemplified by the formation of well supported subclasses within each AFLP clade, which were also observed in the ITS phylogeny. Also, we observed congruence between AFLP and ITS sequences clusters, which was also observed for fungi, oomycetes, plants, and bacteria, indicating that AFLP data can be phylogenetically informative [64], [65], [66]. In our study, the large number of markers analyzed and the high statistical support values obtained showed be due the presence of phylogenetic signals in these data, which has also been observed in other fungi such as *Fusarium oxysporum* [67] and *Verticillium dahliae* [68].

Groups 1 and 2 were also supported by PCR-RFLP techniques using the MspI restriction enzyme for the cleavage of ITS1-5.8S-ITS2 rDNA and BsuRI, PstI or HinfI endonucleases for cleavage of the IGS region, which represents a simple and fast method to differentiate *Epicoccum* isolates. The IGS-RFLP revealed high intraspecific variability: 48 haplotypes were identified in a total of 58 strains. Similar results were observed in *F. oxysporum* [69] and *F. culmorum* [70]. It is known that the occurrence of concerted evolution, by means of unequal chromatid exchange or gene conversion, can lead to homogenization of multiple copies of rDNA region within the individual and the setting of this region can occur in populations of species that reproduce sexually [71]. In this context, in the absence of concerted evolution, multiple variants of the IGS could be expected in the same individual, but this was not observed in our analysis, despite of the fact that two IGS amplification products were obtained for the reference strain CBS 161.73 (not shown). The occurrence of these structural variants within individuals may be explained by a relaxed or low rate of concerted evolution, but further investigation should be carried out to confirm this result for this reference strain.

The techniques based on DNA fingerprinting (AFLP, ITS-RFLP and IGS-RFLP) were able to differentiate isolates and demonstrated the high variability of these strains. These results underscore the importance of using methodologies that differ in the level of resolution to study populations of fungi and obtain a more realistic view of the genetic relationships among isolates [29]. Thus, the results of this study using polyphasic approach including extensive molecular, morphocultural and physiological characterization of the *E. nigrum* contradict previous interpretations that *E. nigrum* is a single variable species. The morphocultural analysis is consistent with earlier descriptions [26], [30], but in the present investigation, we provided further support to separate this morphotypes in distinct species, through molecular analysis.

Although there are recent reviews dealing with phylogenetic affiliations within Dothideomycetes [72], [73], [32], [59], [74], these reports did not analyzed the *Epicoccum* genus consistently. Thus, the present research contributes to the knowledge of this mitosporic genus and asserts that the classification of *E. nigrum* as a single variable species should be reassessed. Based on these data, strains in group 1 correspond to *E. nigrum*, which can be easily identified by the yellow to orange mycelium in PDA, while the strains of group 2, that present variable morphology and form a clade with ITS sequence similarity to *E. andropogonis* and *P. epicoccina* strains, should be reclassified. These findings may have important implications to the development of rational bioprospecting for discovering new bioactive metabolites and more efficient biocontrol strains within natural populations of *Epicoccum*. Further, we suggest that many of the sequences deposited as *E. nigrum* in GenBank and culture collection of microbial strains should be also reclassified, including the reference strain CBS 161.73 sequenced in this work.

## Materials and Methods

### Fungal strains

The *Epicoccum* strains (n = 112) in this study are listed in Table S1. *E. nigrum* strains CBS 318.83 and CBS 161.73 were used as reference for morphological, physiological and genetic characterization. *Epicoccum* was isolated from surface disinfected sugarcane leaves cultivated at the experimental station of Centro de Tecnologia Canavieira – CTC (Piracicaba, São Paulo, Brazil) [6]. Leaves from 36 different plants were sampled in October 2006 (30 months after planting and 60 days after pruning) and immediately carried on to laboratory to fungal isolation. For this, symptomless leaves were washed in running tap water, surface disinfected by serial washing in 70% ethanol for 1 min, sodium hypochlorite (2% of active Cl) for 1.5 min, 70% ethanol for 1 min and 2 rinses in sterilized distilled water. The efficiency of the disinfection process was checked by plating aliquots of the sterile distilled water used in the final rinse on potato dextrose agar (PDA, Merck). After surface disinfection, samples were cut (0.5 cm^2^) and transferred (7 fragments per plate) onto PDA medium amended with tetracycline (50 µg.mL^−1^), and incubated for 5–30 days at 28°C. *Epicoccum-*like isolates were picked out, subcultured to obtain monoconidial colonies and kept at 4°C for further analysis.

### Morphocultural studies

To measure conidia, 46 *Epicoccum* strains (Table S4) were taken from cultures grown on PDA for 20–30 days at 28°C with a 16-h photoperiod. Conidia (n = 85) of each strain were measured in water with an optical microscope (Olympus BH-2, Japan) on a graduated slide (Carl Zeiss). Data of conidial size (length, width and length/width ratio) were subjected to analysis of variance in a completely randomized design (each strain was considered a different treatment). The statistical analysis was carried out with the SAS software (Statistical Analysis System, ©1989–1996 by SAS Institute Inc., Cary, NC, USA) using the Tukey's test (P<0.05) for comparisons of the means. Colony characteristics (color, surface texture and margin shape) of 64 *Epicoccum* strains (Table S2) were evaluated after 10 days of growth at 28°C on Czapeck (Difco), malt (Oxoid), complete [75] and PDA media. The colony diameter in two perpendicular directions was measured every 2 days in each culture medium to determine growth rates and time in lag phase. Tests were performed in triplicate in a completely randomized bifactorial design (strain x culture medium). Each strain was considered a different treatment and analysis of variance was carried out using SAS software. Tukey's test (P<0.05) was conducted to detect significantly different means (Table S2 and Table S3).

### Enzyme assays

To determine extracellular lipase, protease and pectinase activities, a 5-mm culture disc was transferred to culture medium with Tween 20 (lipase), gelatin (protease) or citric pectin (pectin lyase and endopolygalacturonase) as carbon sources [76]. Amylase and endoglucanase production were evaluated on minimal medium [75] with 1% starch (amylase) or 1% carboxymethylcellulose (endoglucanase) instead of glucose as a carbon source. Amylolytic and endoglucanase activities were detected by adding 10 mL of 4% iodine or 5 mL of 0.1% Congo red solution respectively to the fungal colony and were washed with 4M NaCl solution [77]. Starch or carboxymethylcellulose degradation was characterized by the formation of a clear zone surrounding the colony. All analysis was performed in triplicate and evaluated after 4 days of growth. We measured the perpendicular diameters of both the colony and degradation halo: the enzyme index was calculated as the ratio between the halo diameter and colony diameter. Data were subjected to analysis of variance in a completely randomized design (each strain was considered a different treatment). The statistical analysis was carried out with the SAS software (©1989–1996 by SAS Institute Inc., Cary, NC, USA) using the Tukey's test (P<0.05) to detect significantly different means (Table S5).

### Isolation and characterization of Nit mutants

Nit mutants were selected spontaneously on medium containing chlorate, as described previously [78]. Briefly, an agar plug (5-mm) of each monoconidial strain (n = 110, except CBS 318.83 and CBS 161.73 strains) was transferred to medium amended with chlorate (4 plates per strain). Plates were incubated at 28°C for up to 30 days and checked for the emergence of fast-growing sectors. Chlorate-resistant colonies were isolated and purified by the successive transference (at least twice) of the mycelium fragments originating from hyphal tips to fresh chlorate medium. Phenotypic identification (Table S6) and complementation tests among Nit mutants were performed as reported previously [78]. These mutants were used in pairing tests for mycelial interactions.

### Mycelial interactions

*Epicoccum* wild-type strains (n = 50; Table S1) and Nit mutants (n = 32; Table S6) were used to study mycelial interactions. Pairings were conducted by plating agar blocks cut from 2 different strains approximately 2 cm from each other in a 9-cm Petri dish containing PDA medium (for wild-type strains) and nitrate medium (for Nit mutants). Plates were incubated at room temperature (24 to 32°C) for 60 days or until hyphae of both isolates came into contact. Strains were paired in all possible combinations (1225 pairings for wild-type strains, and 496 pairings for Nit mutants) and replicated at least twice. Mycelial reactions were examined from the front and reverse sides of the culture plates. The area of hyphal interaction was photographed for further evaluation.

### DNA extraction, amplification, and sequencing

Monoconidial strains were grown in PD medium for 7 days at 28°C and total DNA was extracted with Wizard Genomic DNA Purification Kit (Promega, USA). We used standard PCR procedures for the amplification of three regions: the internal transcribed spacer (ITS1-5.8S-ITS2) of the rDNA units (570 bp), the β-tubulin gene (350 bp) and the intergenic spacer (IGS) of the rDNA units (2.6 Kb). PCR was performed in 50-µL reactions containing 20 ng of genomic DNA, 0.2 mM dNTPs, 3.7 mM MgCl_2_, 0.8 µM (for ITS) or 0.2 µM (IGS and β-tubulin) of each primer and 2 U of *Taq* DNA polymerase (Fermentas Life Sciences, Brazil) in 1X buffer (5 mM KCl and 2 mM Tris-HCl - pH 8.4). Amplification conditions varied according to the primer set used (Table 3). The amplification products were purified (UltraClean™ PCR Clean-Up Kit, MOBIO Laboratories) and sequenced in both directions using ABI BigDye terminator chemistry on an ABI3700 capillary sequencer at the Laboratory of Molecular Evolution and Bioinformatics, Department of Microbiology (University of São Paulo, Brazil).

**Table 3:** Primer set, target and amplification conditions used in this study.

Primers	5′→3′sequence	Target	Amplification conditions a	Reference
Bt2a	GGTAACCAAATCGGTGCTGCTTTC			
Bt2b	ACCCTCAGTGTAGTGACCCTTGGC	β-tubulin	30 cycles of 94°C for 1 min, 58°C for 1 min and 72°C for 1 min	[91]
CNL12	CTGAACGCCTCTAAGTCAG			
CNS1	GAGACAAGCATATGACTACTG	IGS	30 cycles of 94°C for 1 min, 58°C for 1 min and 72°C for 2 min	[92]
ITS1-F	CTTGGTCATTTAGAGGAAGTAA			
ITS-4	TCCTCCGCTTATTGATATGC	ITS1-5.8S-ITS2	30 cycles of 94°C for 30s, 55°C for 30 s and 72°C for 30 s	[92], [93]

a All amplification reactions were performed with initial denaturation at 94°C for 4 minutes and a final extension at 72°C for 10 minutes.

### Sequence analysis

Complete sequences of the ITS1-5.8S-ITS2 region and partial sequences of the β-tubulin gene from 106 *Epicoccum* strains, including the *E. nigrum* reference strains CBS 318.83 and CBS 161.73 (Table S1) were used for phylogenetic analysis. For these two datasets (n = 106), the ITS and β-tubulin gene sequences of *Phaeosphaeria nodorum* were used as outgroup (AF250830 and AY786336, respectively). ITS region and β-tubulin gene sequences were recovered from GenBank and also included in an additional phylogenetic analysis (Table S7). These sequences include accessions of *Epicoccum* (n = 85, including ATCC strains), *E. andropogonis*, *Phoma epicoccina* ( = *E. nigrum*), and different *Phoma* species that produce dictyochlamydospores and have been phylogenetically related to *P. epicoccina*, as reported previously [32]. In this way, two additional datasets (with 226 taxa for ITS1-5.8S-ITS2 and 135 taxa for β-tubulin) were obtained. For these datasets, the ITS and β-tubulin gene sequences of *P. zantedeschiae* CBS 131.93 and *P. exigua* var. *exigua* CBS 431.74 were used as outgroups (Table S7).

Sequences were aligned by MUSCLE [79] and edited using Se-Al (http://tree.bio.ed.ac.uk/software/seal/). Maximum parsimony and Bayesian analyses were performed for each dataset. The maximum parsimony analysis was conducted using TNT [80] with 100 independent hits to the best score. Afterward the strict consensus on the retained trees was calculated and standard bootstrap analyses with 1000 replicates were performed. Bayesian inference was done with MrBayes [81], using GTR+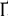
_4_+I for both genes with 10^6^ generations sampling every 1000 generations. The convergence of runs was evaluated using TRACER 1.4 (http://tree.bio.ed.ac.uk/software/tracer). Bayesian posterior probabilities (PP) were obtained from the 50% majority rule consensus after a burn in of 1000 trees. To detect incongruence between ITS and β-tubulin datasets, we performed the partition homogeneity test implemented in PAUP* 4.0b [82] using heuristic search and 1000 replications. The mean pairwise genetic distance and standard error (SE) were calculated for clades obtained in the phylogenetic analysis of the ITS region using MEGA 4.0 [83].

### PCR-RFLP analysis

Polymorphisms in IGS region of the rDNA were evaluated by the PCR-RFLP technique. PCR products were digested with BsuRI, XhoI, BamHI, HincII, ClaI, NheI, EcoRI, Hin6I, MspI, SphI, HinfI, PvuII, RsaI, MboI, PstI, SmaI and EcoRV endonucleases (Fermentas Life Sciences, Brazil) for 3 h at 37°C following the manufacturer's recommendations. The ITS1-5.8S-ITS2 region of rDNA was also evaluated by PCR-RFLP. For this, PCR products were cleaved with BsuRI, HinfI, HhaI, ApaI, PvuII, RsaI, PstI, ClaI, Hin6I, BgIII, BamHI, SpHI, MspI, NheI, EcoRI, NdeI, MboI, TaqI, KpnI and DdeI endonucleases (Fermentas Life Sciences, Brazil). The samples were electrophoresed in 2.4% agarose gels, stained with ethidium bromide and photographed under UV light.

The presence (1) or absence (0) of bands was recorded and the data were analyzed with NTSYS-PC Version 2.1 [84]. Similarity matrices were obtained based on the Dice coefficients using the SIMQUAL program in the software package. Cluster analysis of matrix values was performed by the unweighted pair-group method [85] with arithmetic averages (UPGMA) using the SAHN algorithm [84]. To estimate the strength of the grouping generated by cluster analysis, bootstrap analysis was performed with 1000 replications using WINBOOT [86]. Analysis of the IGS-RFLP data was performed using the restriction site model in MrBayes [81] as described previously [66].

### Characterization of isolates by AFLP analysis

AFLP analysis was performed according to standard methods [87]. Briefly, *E. nigrum* genomic DNA (approximately 200 ng) was digested with 5 U of EcoRI and MseI endonucleases by incubation overnight at 37°C in a 50-µl reaction volume. The reactions were heat-inactivated at 70°C for 10 min and adapters for EcoRI and MseI were linked to the fragments at 23°C for 3 h using T4 DNA ligase. After ligation, two rounds of PCR amplification were performed. The first round was carried out with primers specific for the EcoRI adapters (core primer with an adenine or cytosine as a selective nucleotide) or MseI (core primer with an adenine and a cytosine as selective nucleotides). Amplification was performed for 26 cycles at 94°C for 1 min, annealing at 56°C for 1 min, and extension at 72°C for 1 min. PCR products were diluted five-fold in TE buffer (10 mM Tris–Cl, pH 8.0, 0.1 mM EDTA) and used for amplification with a specific primer for the EcoRI adapter-selective nucleotides (E–A) and to the MseI adapter plus two selective nucleotides (M-CA and M-AT). Selective PCR amplification was as follows: two cycles at 94°C for 30 s, 65°C for 30 s, and 72°C for 1 min. The same conditions for denaturation and extension were maintained for 12 cycles and then the annealing temperature was dropped to 56°C and the extension step was extended to 2 min for 23 cycles. To visualize AFLP patterns, amplification reactions were mixed with an equal volume (8 µL) of formamide dye (98% formamide, 10 mM EDTA, pH 8.0, with bromophenol blue and xylene cyanoll as tracking dyes), heat-inactivated for 5 min at 95°C and chilled on ice. Samples (15 µl) were then loaded on a 6% polyacrylamide gel in 1X TBE buffer with a sensor using 80 W per gel at maximum temperature of 50°C. The AFLP gel was stained with AgNO_3_ [88].

For each primer combination, all bands were manually recorded as present or absent and their sizes were noted. For each of the 55 isolates, the AFLP pattern was described as binary strings coded by the presence (1) or absence (0) of each polymorphic marker. Only clearly repeatable markers between 50 and 500 bp were used for analysis with NTSYS-PC Version 2.1 software as performed by PCR-RFLP analysis. In addition, the genetic structure of the population was examined by analysis of molecular variance (AMOVA) using the Arlequin software package [89]. Phylogenetic analysis of the AFLP data was performed as described above for IGS-RFLP.

### Recombination analysis

AFLP data were also used to assess if the populations are recombining or predominantly clonal. The linkage disequilibrium test was performed using the index of multilocus association I_A_ and the index of association r_d_, which were calculated by the MultiLocus software [90]. These indexes have an expected value of zero if populations are recombining (no association of alleles), while a value greater than zero indicates clonality (association of alleles). To evaluate the significance of the indexes the observed value was compared with the expected value under the null hypothesis of recombination (1000 randomizations). If the observed value is significantly different from the expected value (P<0.05), the null hypothesis of complete panmixia is rejected. We performed the recombination analysis using six different datasets: a dataset with all isolates (n = 55), a dataset with isolates from group 1 (n = 30), a dataset with isolates from group 2 (n = 25), and these same datasets containing only haplotypic data (Table 2). These groups were identified based on the clustering revealed by the phenetic analysis of AFLP data. For each dataset, the recombination analysis was performed considering the isolates as a single population. An additional analysis was also performed considering that the isolates were originated from distinct populations based on the well supported subclasses revealed by the previous phenetic analysis of the AFLP data (Table 2).

### Combined phylogenetic analysis

In addition to the individual-data analysis, we carried out a combined analysis with 55 taxa for the two gene sequences and for DNA fingerprint data. Bayesian inference of combined dataset was done with MrBayes [81], using GTR+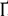
_4_+I for ITS/β-tubulin genes and restriction site model for AFLP/IGS-RFLP data. The search was conducted for 10^7^ generations, sampling every 1000 generations. The convergence of runs was evaluated using TRACER 1.4 (available from http://tree.bio.ed.ac.uk/software/tracer). Maximum clade credibility trees were selected using TreeAnnotator v1.4.7 (http://beast.bio.ed.ac.uk).

## Supporting Information

Table S1 *Epicoccum* strains analyzed in this study.
(0.18 MB DOC)

Table S2 Morphocultural characterization of 64 *Epicoccum* strains in different culture media.
(0.13 MB DOC)

Table S3 Effect of culture media on mycelial growth and lag phase duration of 64 *Epicoccum* strains.
(0.03 MB DOC)

Table S4 Conidial dimensions of 46 *Epicoccum* strains grown on PDA medium.
(0.06 MB DOC)

Table S5 Production of extracellular hydrolytic enzymes on solid media by 64 *Epicoccum* strains.
(0.12 MB DOC)

Table S6 Phenotypic characterization of *Epicoccum* chlorate-resistant mutants.
(0.11 MB DOC)

Table S7 GenBank accession numbers of ITS1-5.8S-ITS2 and β-tubulin sequences used in this study.
(0.10 MB DOC)

Figure S1 Bayesian maximum clade credibility tree inferred using ITS1-5.8S-ITS2 sequences of 106 *Epicoccum* strains. Two *E. nigrum* reference strains (CBS 318.83 and CBS 161.73) were included. *P. nodorum* access AF250830 was used as outgroup. The posterior probability values are shown next to relevant nodes. Orange lines represent the *Epicoccum* strains from group 1. Blue and black lines represent the strains from group 2.
(0.56 MB TIF )

Figure S2 Bayesian maximum clade credibility tree inferred using ITS1-5.8S-ITS2 sequences of 226 taxa, including 106 *Epicoccum* strains in the present study and other accessions of *Epicoccum* and *Phoma* from GenBank. The ITS gene sequences of *P. zantedeschiae* CBS 131.93 and *P. exigua* var. *exigua* CBS 431.74 were used as outgroups. The posterior probability values are shown next to relevant nodes. Strains from group 1 in this study are indicated in orange color, and strains from group 2 in this study are indicated in blue and black colors. Accessions from GenBank are typed in gray.
(3.24 MB TIF)

Figure S3 Bayesian maximum clade credibility tree inferred using {lower case beta}-tubulin partial sequences of 106 *Epicoccum* strains, including two *E. nigrum* reference strains (CBS 318.83 and CBS 161.73). *P. nodorum* access AY786336 was used as outgroup. The posterior probability values are shown next to relevant nodes. Orange lines represent the *Epicoccum* strains from group 1. Blue and black lines represent the strains from group 2.
(0.57 MB TIF)

Figure S4 Bayesian maximum clade credibility tree inferred using concatenated ITS1-5.8S-ITS2 and β-tubulin sequences of 106 *Epicoccum* strains, including two *E. nigrum* reference strains (CBS 318.83 and CBS 161.73). *P. nodorum* accessions AF250830 and AY786336 were used as outgroup. The posterior probability values are shown next to relevant nodes. Orange lines represent the *Epicoccum* strains from group 1. Blue and black lines represent the strains from group 2.
(0.50 MB TIF)

Figure S5 Bayesian maximum clade credibility tree inferred using β-tubulin partial sequences of 135 taxa, including 106 *Epicoccum* strains in the present study and other accessions of *Phoma* from GenBank. The β-tubulin gene sequences of *P. zantedeschiae* CBS 131.93 and *P. exigua* var. *exigua* CBS 431.74 were used as outgroups. The posterior probability values are shown next to relevant nodes. Strains from group 1 in this study are indicated in orange color, and strains from group 2 in this study are indicated in blue and black colors. Accessions from GenBank are typed in gray.
(0.65 MB TIF)

Figure S6 Phenogram derived from clustering analysis (UPGMA) of AFLP data of 55 *Epicoccum* endophytic strains based on the Dice coefficient. Bar represents the similarity coefficient. Bootstrap = 1000.
(0.01 MB PDF)

Figure S7 Example of the digestion profiles (with BsuRI endonuclease) resulting from the cleavage of the intergenic spacer (IGS) region of the rDNA of some *Epicoccum* endophytic isolates. The vertical lines on the left and right sides of the agarose gel represent the 1 Kb and 100 bp molecular weight markers, respectively.
(0.12 MB DOC)

Figure S8 Phenogram derived from clustering analysis (UPGMA) of IGS-RFLP data of 58 *Epicoccum* endophytic strains based on the Dice coefficient. Bar represents the similarity coefficient. Bootstrap = 1000.
(0.01 MB PDF)
