# Supplementary material for: Polyphasic Analysis of Intraspecific Diversity in Epicoccum nigrum Warrants Reclassification into Separate Species
Source: PLoS One. 2011 Aug 11;6(8):e14828. doi: 10.1371/journal.pone.0014828 (PMC3154903; doi:10.1371/journal.pone.0014828)
Supplement: Table S1 — Epicoccum strains analyzed in this study. (0.18 MB DOC) [file pone.0014828.s001.doc]

**Table S1. *Epicoccum* strains analyzed in this study.**

|  |  |  |  | **GenBank accession nº** | | **Analysis performed in this study *^c^*** |  | | | | | |
| --- | --- | --- | --- | --- | --- | --- | --- | --- | --- | --- | --- | --- |
| **Strain** | **Host/Source *^a^*** | **Country/Province** | **Reference** | **ITS1-5.8S-ITS2** | **β-tubulin** | **Morphometric** | **Morphocultural** | **Enzyme assays** | **Mycelial reaction assay** | **AFLP** | **ITS-RFLP** | **IGS-RFLP** |
| CBS 161.73 | *Zea mays* | U.S.A. | CBS *^b^* | GU014950 | GU563404 | - | + | + | - | - | + | - |
| CBS 318.83 | Soil | - | CBS *^b^* | GU014946 | GU563403 | - | + | + | + | - | + | + |
| 1F15 | *Vitis labrusca* leaves | Brazil (Jundiaí, SP) | 2 | GU014945 | GU563387 | + | + | + | + | + | + | + |
| Ep1sc | Caryopses of sugarcane hybrids | Brazil (Camamú, BA) | Martins TD*^d^* | HM999858 | GU563405 | + | + | + | - | + | + | + |
| TH1 | Transgenic sugarcane leaves | Brazil (Piracicaba, SP) | This study | HM999862 | GU563420 | + | + | + | - | + | + | + |
| TH2 | Transgenic sugarcane leaves | Brazil (Piracicaba, SP) | This study | HM999863 | GU563421 | + | + | + | - | + | + | + |
| TC41 | Transgenic sugarcane leaves | Brazil (Piracicaba, SP) | This study | GU014967 | GU563410 | + | + | + | + | + | + | + |
| P16 | Non-transgenic sugarcane leaves | Brazil (Piracicaba, SP) | This study | GU014948 | GU563381 | + | + | + | + | + | + | + |
| P17 | Non-transgenic sugarcane leaves | Brazil (Piracicaba, SP) | This study | HM999853 | GU563382 | + | + | + | + | + | + | + |
| P18 | Non-transgenic sugarcane leaves | Brazil (Piracicaba, SP) | This study | GU014951 | GU563428 | + | + | + | - | + | + | + |
| CE5 | Non-transgenic sugarcane leaves | Brazil (Piracicaba, SP) | This study | GU014930 | GU563393 | + | + | + | + | + | + | + |
| CE9 | Non-transgenic sugarcane leaves | Brazil (Piracicaba, SP) | This study | GU014932 | GU563396 | + | + | + | + | + | + | + |
| CE11 | Non-transgenic sugarcane leaves | Brazil (Piracicaba, SP) | This study | GU014934 | HM999799 | + | + | + | + | + | + | + |
| CE12 | Non-transgenic sugarcane leaves | Brazil (Piracicaba, SP) | This study | GU014935 | HM999800 | + | + | + | + | + | + | + |
| CE13 | Non-transgenic sugarcane leaves | Brazil (Piracicaba, SP) | This study | GU014936 | HM999801 | + | + | + | - | + | + | + |
| CE16 | Non-transgenic sugarcane leaves | Brazil (Piracicaba, SP) | This study | GU014937 | GU563398 | + | + | + | + | + | + | + |
| CE18 | Non-transgenic sugarcane leaves | Brazil (Piracicaba, SP) | This study | GU014938 | GU563399 | + | + | + | + | + | + | + |
| CE24 | Non-transgenic sugarcane leaves | Brazil (Piracicaba, SP) | This study | HM999857 | HM999802 | + | + | + | - | + | + | + |
| CE25 | Non-transgenic sugarcane leaves | Brazil (Piracicaba, SP) | This study | GU014939 | GU563400 | + | + | + | - | + | + | + |
| CE27 | Non-transgenic sugarcane leaves | Brazil (Piracicaba, SP) | This study | GU014940 | GU563401 | + | + | + | + | + | + | + |
| CE29 | Non-transgenic sugarcane leaves | Brazil (Piracicaba, SP) | This study | GU014941 | HM999803 | + | + | + | - | + | + | + |
| C41A | Non-transgenic sugarcane leaves | Brazil (Piracicaba, SP) | This study | GU014985 | GU563423 | + | + | + | + | + | + | + |
| C41B | Non-transgenic sugarcane leaves | Brazil (Piracicaba, SP) | This study | GU014986 | GU563424 | + | + | + | + | + | + | + |
| C22B | Non-transgenic sugarcane leaves | Brazil (Piracicaba, SP) | This study | GU014983 | GU563417 | + | + | + | + | + | + | + |
| 1F4 | *Vitis labrusca* leaves | Brazil (Jundiaí, SP) | 2 | GU014943 | GU563386 | - | + | + | + | + | + | + |
| EpAr | *Ricinus communis* leaves | Brazil (Lavras, MG) | Castro RA*^d^* | HM999851 | GU563385 | - | + | + | - | + | + | + |
| 63Ep | Transgenic sugarcane leaves | Brazil (Piracicaba, SP) | 3 | GU014923 | HM999794 | - | + | + | + | + | + | + |
| 79Ep | Transgenic sugarcane rizosphere | Brazil (Piracicaba, SP) | 3 | GU014924 | GU563390 | - | + | + | + | + | + | + |
| TH21Ep | Transgenic sugarcane leaves | Brazil (Piracicaba, SP) | This study | GU014927 | GU563391 | - | + | + | + | + | + | + |
| TC1 | Transgenic sugarcane leaves | Brazil (Piracicaba, SP) | This study | HM999860 | GU563422 | - | + | + | + | + | + | + |
| TC42A | Transgenic sugarcane leaves | Brazil (Piracicaba, SP) | This study | GU014969 | GU563408 | - | + | + | + | + | + | + |
| TC42F | Transgenic sugarcane leaves | Brazil (Piracicaba, SP) | This study | GU014971 | GU563409 | - | + | + | - | + | + | + |
| TH31B | Transgenic sugarcane leaves | Brazil (Piracicaba, SP) | This study | GU014974 | GU563429 | - | + | + | + | + | + | + |
| SP1 | Non-transgenic sugarcane leaves | Brazil (Piracicaba, SP) | This study | GU014966 | GU563412 | - | + | + | + | + | + | + |
| P11 | Non-transgenic sugarcane leaves | Brazil (Piracicaba, SP) | This study | HM999852 | GU563380 | - | + | + | - | + | + | + |
| P12 | Non-transgenic sugarcane leaves | Brazil (Piracicaba, SP) | This study | GU014947 | HM999796 | - | + | + | + | + | + | + |
| C33Ep | Non-transgenic sugarcane leaves | Brazil (Piracicaba, SP) | This study | GU014925 | GU563388 | - | + | + | + | + | + | + |
| CE2 | Non-transgenic sugarcane leaves | Brazil (Piracicaba, SP) | This study | GU014929 | GU563392 | - | + | + | + | + | + | + |
| CE6 | Non-transgenic sugarcane leaves | Brazil (Piracicaba, SP) | This study | HM999855 | HM999798 | - | + | + | + | + | + | + |
| CE7 | Non-transgenic sugarcane leaves | Brazil (Piracicaba, SP) | This study | HM999856 | GU563395 | - | + | + | - | + | + | + |
| CE10 | Non-transgenic sugarcane leaves | Brazil (Piracicaba, SP) | This study | GU014933 | GU563397 | - | + | + | + | + | + | + |
| CE39 | Non-transgenic sugarcane leaves | Brazil (Piracicaba, SP) | This study | GU014942 | GU563402 | - | + | + | + | + | + | + |
| CV3 | Non-transgenic sugarcane leaves | Brazil (Piracicaba, SP) | This study | GU014984 | GU563419 | - | + | + | + | + | + | + |
| C12A | Non-transgenic sugarcane leaves | Brazil (Piracicaba, SP) | This study | GU014977 | GU563416 | - | + | + | + | + | + | + |
| C13A | Non-transgenic sugarcane leaves | Brazil (Piracicaba, SP) | This study | GU014980 | GU563414 | - | + | + | + | + | + | + |
| C42A | Non-transgenic sugarcane leaves | Brazil (Piracicaba, SP) | This study | GU014987 | GU563425 | - | + | + | - | + | + | + |
| P98 | Non-transgenic sugarcane leaves | Brazil (Piracicaba, SP) | This study | GU014949 | GU563383 | - | + | + | + | + | + | + |
| C42B | Non-transgenic sugarcane leaves | Brazil (Piracicaba, SP) | This study | GU014988 | GU563426 | - | - | - | - | + | + | + |
| P14 | Non-transgenic sugarcane leaves | Brazil (Piracicaba, SP) | This study | HM999865 | GU563427 | - | - | - | - | + | + | + |
| TC41F | Transgenic sugarcane leaves | Brazil (Piracicaba, SP) | This study | GU014968 | GU563411 | - | - | - | - | + | + | + |
| Ep1m | *Malus domestica* leaves | Brazil (Caxias do Sul, RS) | 1 | HM999850 | GU563384 | - | - | - | - | + | + | + |
| Ep2sc | Caryopses of sugarcane hybrids | Brazil (Camamú, BA) | Martins TD*^d^* | HM999859 | GU563406 | - | - | - | - | + | + | + |
| C41Ep | Non-transgenic sugarcane leaves | Brazil (Piracicaba, SP) | This study | GU014926 | GU563389 | - | - | - | - | + | + | + |
| SP2 | Non-transgenic sugarcane leaves | Brazil (Piracicaba, SP) | This study | HM999861 | GU563413 | + | + | + | + | + | + | + |
| CE51* | Non-transgenic sugarcane leaves | Brazil (Piracicaba, SP) | This study | GU014931 | GU563394 | + | + | + | + | + | + | - |
| CV2 | Non-transgenic sugarcane leaves | Brazil (Piracicaba, SP) | This study | GU014982 | GU563418 | + | + | + | + | + | + | + |
| C13B | Non-transgenic sugarcane leaves | Brazil (Piracicaba, SP) | This study | GU014981 | GU563415 | + | + | + | + | + | + | + |
| 62Ep | Transgenic sugarcane leaves | Brazil (Piracicaba, SP) | 3 | GU014976 | GU563407 | - | + | + | + | + | + | + |
| TH31A | Transgenic sugarcane leaves | Brazil (Piracicaba, SP) | This study | GU014973 | HM999808 | - | + | + | + | - | - | + |
| C12B | Non-transgenic sugarcane leaves | Brazil (Piracicaba, SP) | This study | GU014978 | HM999804 | - | + | + | - | - | - | + |
| 1F6 | *Vitis labrusca* leaves | Brazil (Jundiaí, SP) | 2 | GU014944 | HM999793 | - | + | + | - | - | - | - |
| TH41Ep | Transgenic sugarcane leaves | Brazil (Piracicaba, SP) | This study | GU014928 | HM999795 | - | + | + | + | - | - | - |
| TC2 | Transgenic sugarcane leaves | Brazil (Piracicaba, SP) | This study | GU014975 | HM999806 | - | + | + | + | - | - | - |
| TH13F* | Transgenic sugarcane leaves | Brazil (Piracicaba, SP) | This study | GU014972 | ND | - | + | + | - | - | - | - |
| CE3 | Non-transgenic sugarcane leaves | Brazil (Piracicaba, SP) | This study | HM999854 | HM999797 | - | + | + | + | - | - | - |
| CE22 | Non-transgenic sugarcane leaves | Brazil (Piracicaba, SP) | This study | ND | ND | - | + | + | - | - | - | - |
| CV1 | Non-transgenic sugarcane leaves | Brazil (Piracicaba, SP) | This study | ND | ND | - | + | + | + | - | - | - |
| C12C | Non-transgenic sugarcane leaves | Brazil (Piracicaba, SP) | This study | GU014979 | HM999805 | - | + | + | + | - | - | - |
| C22A | Non-transgenic sugarcane leaves | Brazil (Piracicaba, SP) | This study | ND | ND | - | + | + | - | - | - | - |
| P13 | Non-transgenic sugarcane leaves | Brazil (Piracicaba, SP) | This study | HM999864 | HM999809 | + | + | + | + | - | - | - |
| P21 | Transgenic sugarcane leaves | Brazil (Piracicaba, SP) | This study | GU014953 | HM999811 | + | - | - | - | - | - | - |
| P24 | Transgenic sugarcane leaves | Brazil (Piracicaba, SP) | This study | GU014954 | HM999813 | + | - | - | - | - | - | - |
| P31 | Transgenic sugarcane leaves | Brazil (Piracicaba, SP) | This study | GU014955 | HM999814 | + | - | - | - | - | - | - |
| P42* | Transgenic sugarcane leaves | Brazil (Piracicaba, SP) | This study | GU014959 | ND | + | - | - | - | - | - | - |
| P44 | Transgenic sugarcane leaves | Brazil (Piracicaba, SP) | This study | HM999866 | HM999819 | + | - | - | - | - | - | - |
| P51 | Non-transgenic sugarcane leaves | Brazil (Piracicaba, SP) | This study | GU014960 | HM999820 | + | - | - | - | - | - | - |
| P55 | Non-transgenic sugarcane leaves | Brazil (Piracicaba, SP) | This study | GU014963 | HM999823 | + | - | - | - | - | - | - |
| P58 | Non-transgenic sugarcane leaves | Brazil (Piracicaba, SP) | This study | HM999867 | HM999825 | + | - | - | - | - | - | - |
| P61 | Transgenic sugarcane leaves | Brazil (Piracicaba, SP) | This study | HM999868 | HM999826 | + | - | - | - | - | - | - |
| P62 | Transgenic sugarcane leaves | Brazil (Piracicaba, SP) | This study | HM999869 | HM999827 | + | - | - | - | - | - | - |
| P64 | Transgenic sugarcane leaves | Brazil (Piracicaba, SP) | This study | GU014965 | HM999828 | + | - | - | - | - | - | - |
| P74 | Transgenic sugarcane leaves | Brazil (Piracicaba, SP) | This study | GU015005 | HM999830 | + | - | - | - | - | - | - |
| P81 | Transgenic sugarcane leaves | Brazil (Piracicaba, SP) | This study | GU015007 | HM999832 | + | - | - | + | - | - | - |
| P82 | Transgenic sugarcane leaves | Brazil (Piracicaba, SP) | This study | GU015008 | HM999833 | + | - | - | + | - | - | - |
| P83 | Transgenic sugarcane leaves | Brazil (Piracicaba, SP) | This study | GU015009 | HM999834 | + | - | - | - | - | - | - |
| P910 | Non-transgenic sugarcane leaves | Brazil (Piracicaba, SP) | This study | GU014995 | HM999841 | + | - | - | - | - | - | - |
| P91 | Non-transgenic sugarcane leaves | Brazil (Piracicaba, SP) | This study | HM999870 | HM999835 | + | - | - | - | - | - | - |
| P92 | Non-transgenic sugarcane leaves | Brazil (Piracicaba, SP) | This study | GU014990 | HM999836 | + | - | - | + | - | - | - |
| P112 | Transgenic sugarcane leaves | Brazil (Piracicaba, SP) | This study | GU015002 | HM999848 | + | - | - | - | - | - | - |
| TC42B | Transgenic sugarcane leaves | Brazil (Piracicaba, SP) | This study | GU014970 | HM999807 | - | - | - | - | - | - | - |
| P19 | Non-transgenic sugarcane leaves | Brazil (Piracicaba, SP) | This study | GU014952 | HM999810 | - | - | - | - | - | - | - |
| P22 | Non-transgenic sugarcane leaves | Brazil (Piracicaba, SP) | This study | GU014989 | HM999812 | - | - | - | - | - | - | - |
| P32 | Transgenic sugarcane leaves | Brazil (Piracicaba, SP) | This study | GU014957 | HM999815 | - | - | - | - | - | - | - |
| P33 | Transgenic sugarcane leaves | Brazil (Piracicaba, SP) | This study | GU014958 | HM999816 | - | - | - | + | - | - | - |
| P34 | Non-transgenic sugarcane leaves | Brazil (Piracicaba, SP) | This study | GU014998 | HM999817 | - | - | - | - | - | - | - |
| P311 | Transgenic sugarcane leaves | Brazil (Piracicaba, SP) | This study | GU014956 | HM999818 | - | - | - | - | - | - | - |
| P52 | Non-transgenic sugarcane leaves | Brazil (Piracicaba, SP) | This study | GU014961 | HM999821 | - | - | - | - | - | - | - |
| P54 | Non-transgenic sugarcane leaves | Brazil (Piracicaba, SP) | This study | GU014962 | HM999822 | - | - | - | - | - | - | - |
| P57 | Non-transgenic sugarcane leaves | Brazil (Piracicaba, SP) | This study | GU014964 | HM999824 | - | - | - | - | - | - | - |
| P71 | Transgenic sugarcane leaves | Brazil (Piracicaba, SP) | This study | GU015004 | HM999829 | - | - | - | - | - | - | - |
| P75 | Transgenic sugarcane leaves | Brazil (Piracicaba, SP) | This study | GU015006 | HM999831 | - | - | - | - | - | - | - |
| P93 | Non-transgenic sugarcane leaves | Brazil (Piracicaba, SP) | This study | GU014991 | HM999837 | - | - | - | - | - | - | - |
| P96 | Non-transgenic sugarcane leaves | Brazil (Piracicaba, SP) | This study | GU014992 | HM999838 | - | - | - | - | - | - | - |
| P97 | Non-transgenic sugarcane leaves | Brazil (Piracicaba, SP) | This study | GU014993 | HM999839 | - | - | - | - | - | - | - |
| P99 | Non-transgenic sugarcane leaves | Brazil (Piracicaba, SP) | This study | GU014994 | HM999840 | - | - | - | - | - | - | - |
| P911 | Non-transgenic sugarcane leaves | Brazil (Piracicaba, SP) | This study | GU014996 | HM999842 | - | - | - | - | - | - | - |
| P912 | Non-transgenic sugarcane leaves | Brazil (Piracicaba, SP) | This study | GU014997 | HM999843 | - | - | - | + | - | - | - |
| P102 | Transgenic sugarcane leaves | Brazil (Piracicaba, SP) | This study | GU014999 | HM999844 | - | - | - | - | - | - | - |
| P103 | Transgenic sugarcane leaves | Brazil (Piracicaba, SP) | This study | GU015000 | HM999845 | - | - | - | - | - | - | - |
| P104 | Transgenic sugarcane leaves | Brazil (Piracicaba, SP) | This study | GU015001 | HM999846 | - | - | - | - | - | - | - |
| P111 | Transgenic sugarcane leaves | Brazil (Piracicaba, SP) | This study | GU015010 | HM999847 | - | - | - | - | - | - | - |
| P121 | Non-transgenic sugarcane leaves | Brazil (Piracicaba, SP) | This study | GU015003 | HM999849 | - | - | - | - | - | - | - |

*^a^* Transgenic sugarcane corresponds to variety SP80-1842 IMI-01; Non-transgenic sugarcane corresponds to variety SP80-1842. *^b^* Reference strains; CBS, Centraalbureau voor Schimmelcultures, Utrecht, Netherlands. *^c^* A plus sign (+) indicates that the test was performed; A minus sign (-) indicates that the test was not performed. *^d^* Personal communication. (ND) Not determined. (*) The strains CE51, TH13F and P42 were not included in the phylogenetic analysis.

**Reference**

1. Camatti-Sartori V, Azevedo JL, Sanhueza RMV, Ribeiro RTS, Echeverrigaray S (2005) Endophytic yeasts and filamentous fungi associated with southern Brazilian apple (*Malus domestica*) orchards subjected to conventional, integrated or organic cultivation. J Basic Microbiol 45: 397-402.

2. Brum MCP (2008) Potencial biotecnológico de fungos endofíticos da videira. Phd Thesis, Universidade de Mogi das Cruzes.

3. Romão AS, Araújo WL (2007) Efeito do cultivo de cana-de-açúcar geneticamente modificada sobre a comunidade fúngica associada. *In* L. Costa-Maia, E. Malosso, A. M. Yano-Melo (eds.), Micologia: avanços no conhecimento. 659 Recife: Editora Universitária da UFPE, p. 150-159.
