# Supplementary material for: Polyphasic Analysis of Intraspecific Diversity in Epicoccum nigrum Warrants Reclassification into Separate Species
Source: PLoS One. 2011 Aug 11;6(8):e14828. doi: 10.1371/journal.pone.0014828 (PMC3154903; doi:10.1371/journal.pone.0014828)
Supplement: Table S2 — Morphocultural characterization of 64 Epicoccum strains in different culture media. (0.13 MB DOC) [file pone.0014828.s002.doc]

**Table S2. Morphocultural characterization of 64 *Epicoccum* strains in different culture media.**

|  |  |  |  |  | **Growth characteristics in different culture media *^d^*** | | | |
| --- | --- | --- | --- | --- | --- | --- | --- | --- |
| **Strain** | **ITS group*^a^*** | **AFLP group*^b^*** | **Growth *^c^* (cm.day^-1^)** | **Lag phase *^c^* (hours)** | **PDA** | **Malt** | **Czapeck** | **Complete medium** |
| TH31A | 2 | ND | 1.44 ^a^ | 3.70 ^ab^ | sup., dense, unif.; dark pink, rev. purple | sup., dense, unif.; dark pink, rev. purple | sup., sparse, unif.; pale ye., rev. pale ye. | sup., sparse, unif.; pale ye., rev. pale ye. |
| 62Ep | 2 | 2A | 1.42 ^a^ | 8.37 ^zwyvuatx^ | sup., dense, unif.; grey, rev. dark grey | sup., dense, unif.; dark pink, rev. purple | sup., sparse, unif.; pale ye., rev. pale ye. | sup., sparse, unif.; pale ye., rev. pale ye. |
| SP1 | 2 | 2A | 1.37 ^ba^ | 13.62 ^nmsqptro^ | sup., dense, unif.; grey, rev. dark grey | sup., dense, unif.; dark pink, rev. purple | sup., sparse, unif.; pale ye., rev. pale ye. | sup., sparse, unif.; pale ye., rev. pale ye. |
| C12A | 2 | 2A | 1.37 ^ba^ | 10.77 ^wvusqptrx^ | sup., dense, unif.; grey, rev. dark grey | sup., dense, unif.; dark pink, rev. purple | sup., sparse, unif.; pale ye., rev. pale ye. | sup., sparse, unif.; pale ye., rev. pale ye. |
| CV3 | 2 | 2B | 1.31 ^bac^ | 4.95 ^zyabx^ | sup., dense, unif.; grey, rev. dark grey | sup., dense, unif.; dark pink, rev. purple | sup., sparse, unif.; pale ye., rev. pale ye. | sup., sparse, unif.; pale ye., rev. pale ye. |
| CV2 | 2 | 2B | 1.28 ^bdac^ | 5.22 ^zyabx^ | sup., dense, unif.; grey, rev. dark grey | sup., dense, unif.; dark pink, rev. purple | sup., sparse, unif.; pale ye., rev. pale ye. | sup., sparse, unif.; pale ye., rev. pale ye. |
| C41A | 2 | 2B | 1.27 ^bdac^ | 7.83 ^zwyvuatbx^ | sup., dense, unif.; grey, rev. dark grey | sup., dense, unif.; dark pink, rev. purple | sup., sparse, unif.; pale ye., rev. pale ye. | sup., sparse, unif.; pale ye., rev. pale ye. |
| C22B | 2 | 2B | 1.26 ^ebdac^ | 5.09 ^zyabx^ | sup., dense, unif.; grey, rev. dark grey | sup., dense, unif.; dark pink, rev. purple | sup., sparse, unif.; pale ye., rev. pale ye. | sup., sparse, unif.; pale ye., rev. pale ye. |
| TC42A | 2 | 2A | 1.26 ^ebdac^ | 9.37 ^zwyvusqatrx^ | sup., dense, unif.; pink., rev. pink | sup., dense, unif.; pink., rev. pink | sup., sparse, unif.; pale ye., rev. pale ye. | sup., sparse, unif.; pale ye., rev. pale ye. |
| C22A | 2 | ND | 1.25 ^ebdac^ | 5.77 ^zwyabx^ | sup., dense, unif.; dark pink, rev. purple | sup., dense, unif.; dark pink, rev. purple | sup., sparse, unif.; pale ye., rev. pale ye. | sup., sparse, unif.; pale ye., rev. pale ye. |
| C13B | 2 | 2A | 1.24 ^ebacf^ | 8.94 ^zwyvusatx^ | sup., dense, unif.; grey, rev. dark grey | sup., dense, unif.; dark pink, rev. purple | sup., sparse, unif.; pale ye., rev. pale ye. | sup., sparse, unif.; pale ye., rev. pale ye. |
| TH13F | 2 | ND | 1.21 ^ebdgcf^ | 5.12 ^zyabx^ | sup., dense, unif.; dark pink, rev. purple | sup., dense, unif.; dark pink, rev. purple | sup., sparse, unif.; pale ye., rev. pale ye. | sup., sparse, unif.; pale ye., rev. pale ye. |
| C13A | 2 | 2A | 1.19 ^ebdghcf^ | 7.90 ^zwyvuatbx^ | sup., dense, unif.; grey, rev. dark grey | sup., dense, unif.; dark pink, rev. purple | sup., sparse, unif.; pale ye., rev. pale ye. | sup., sparse, unif.; pale ye., rev. pale ye. |
| TC41 | 2 | 2A | 1.17 ^ebdighcf^ | 8.36 ^zwyvuatx^ | sup., dense, unif.; dark pink, rev. purple | sup., dense, unif.; dark pink, rev. purple | sup., sparse, unif.; pale ye., rev. pale ye. | sup., sparse, unif.; pale ye., rev. pale ye. |
| P13 | 2 | ND | 1.17 ^ebdighcf^ | 7.02 ^zwyvabx^ | sup., dense, unif.; grey, rev. dark grey | sup., dense, unif.; dark pink, rev. purple | sup., sparse, unif.; pale ye., rev. pale ye. | sup., sparse, unif.; pale ye., rev. pale ye. |
| TH31B | 2 | 2A | 1.15 ^ejdighcf^ | 4.56 ^zab^ | sup., dense, unif.; dark pink, rev. purple | sup., dense, unif.; dark pink, rev. purple | sup., sparse, unif.; pale ye., rev. pale ye. | sup., sparse, unif.; pale ye., rev. pale ye. |
| TC42F | 2 | 2A | 1.13 ^eljkdighcf^ | 9.10 ^zwyvusatrx^ | sup., dense, unif.; pink., rev. pink. | sup., dense, unif.; pink., rev. pink | sup., sparse, unif.; pale ye., rev. pale ye. | sup., sparse, unif.; pale ye., rev. pale ye. |
| SP2 | 2 | 2A | 1.10 ^eljkdighmf^ | 5.79 ^zwyabx^ | sup., dense, unif.; grey, rev. dark grey | sup., dense, unif.; dark pink, rev. purple | sup., sparse, unif.; pale ye., rev. pale ye. | sup., sparse, unif.; pale ye., rev. pale ye. |
| TH2 | 2 | 2A | 1.07 ^eljkighmf^ | 4.85 ^zyab^ | sup., dense, unif.; grey, rev. dark grey | sup., dense, unif.; dark pink, rev. purple | sup., sparse, unif.; pale ye., rev. pale ye. | sup., sparse, unif.; pale ye., rev. pale ye. |
| TH1 | 2 | 2A | 1.05 ^ljknighmf^ | 3.74 ^ab^ | sup., dense, unif.; grey, rev. dark grey | sup., dense, unif.; dark pink, rev. purple | sup., sparse, unif.; pale ye., rev. pale ye. | sup., sparse, unif.; pale ye., rev. pale ye. |
| C41B | 2 | 2A | 1.05 ^ljknighm^ | 6.43 ^zwyvabx^ | sup., dense, unif.; grey, rev. dark grey | sup., dense, unif.; dark pink, rev. purple | sup., sparse, unif.; pale ye., rev. pale ye. | sup., sparse, unif.; pale ye., rev. pale ye. |
| TC1 | 2 | 2A | 1.04 ^oljknighm^ | 7.74 ^zwyvuabx^ | sup., dense, unif.; pink., rev. pink | sup., dense, unif.; pink., rev. pink | sup., sparse, unif.; pale ye., rev. pale ye. | sup., sparse, unif.; pale ye., rev. pale ye. |
| P18 | 2 | 2B | 1.02 ^oljknihmp^ | 2.33 ^b^ | sup., dense, unif.; grey, rev. dark grey | sup., dense, unif.; dark pink, rev. purple | sup., sparse, unif.; pale ye., rev. pale ye. | sup., sparse, unif.; pale ye., rev. pale ye. |
| C42A | 2 | 2A | 0.98 ^oljkniqmp^ | 7.84 ^zwyvuatbx^ | sup., dense, unif.; dark pink, rev. purple | sup., dense, unif.; dark pink, rev. purple | sup., sparse, unif.; pale ye., rev. pale ye. | sup., sparse, unif.; pale ye., rev. pale ye. |
| TC2 | 2 | ND | 0.96 ^olrknqmp^ | 6.28 ^zwyvabx^ | sup., dense, unif.; dark pink, rev. purple | sup., dense, unif.; dark pink, rev. purple | sup., sparse, unif.; pale ye., rev. pale ye. | sup., sparse, unif.; pale ye., rev. pale ye. |
| C12C | 2 | ND | 0.84 ^ortqsup^ | 8.92 ^zwyvusatx^ | sup., dense, unif.; pink., rev. pink | sup., dense, unif.; pink., rev. pink | sup., sparse, unif.; pale ye., rev. pale ye. | sup., sparse, unif.; pale ye., rev. pale ye. |
| Ep1sc | 2 | 2C | 0.83 ^rvtqsup^ | 19.23 ^gmkiljh^ | sup., dense, irreg.; pale gray, rev. grey | sup., dense, irreg.; pale gray, rev. grey | sup., dense, irreg.; dark grey, rev. dark grey | sup., dense, irreg.; grey, rev. grey |
| CV1 | 2 | ND | 0.77 ^wrvtxsu^ | 4.45 ^ab^ | sup., dense, unif.; grey, rev. dark grey | sup., dense, unif.; dark pink, rev. purple | sup., sparse, unif.; pale ye., rev. pale ye. | sup., sparse, unif.; pale ye., rev. pale ye. |
| C12B | 2 | ND | 0.70 ^wyvxu^ | 2.147 ^b^ | sup., dense, unif.; dark pink, rev. purple | sup., dense, unif.; dark pink, rev. purple | sup., sparse, unif.; pale ye., rev. pale ye. | sup., sparse, unif.; pale ye., rev. pale ye. |
| CBS 161.73 ***^e^*** | 2 | ND | 0.32 ^c^ | 36.63 ^a^ | sup., dense, irreg., pale pink, rev. dark pink | sup., dense, irreg., pale pink, rev. dark pink | sup., dense, irreg., pale pink, rev. grey | sup., dense, irreg., pale pink, rev. grey |
| TH41Ep | 1 | ND | 1.31 ^bac^ | 31.49 ^ba^ | aerial, dense, irreg.; or., rev. or.-red. | aerial, dense, irreg.; or., rev. or.-red. | sup., sparse, unif.; pale ye., rev. ye. | sup., dense, irreg.; ye., rev. or.-bro. |
| C33Ep | 1 | 1A | 1.19 ^ebdghcf^ | 28.67 ^bc^ | aerial, dense, irreg.; or., rev. or.-red. | aerial, dense, irreg.; or., rev. or.-red. | sup., sparse, unif.; pale ye., rev. ye. | sup., dense, irreg.; ye., rev. or.-bro. |
| P17 | 1 | 1A | 1.14 ^ejkdighcf^ | 27.58 ^bcd^ | aerial, dense, irreg.; or., rev. or.-red. | aerial, dense, irreg.; or., rev. or.-red. | sup., sparse, unif.; pale ye., rev. ye. | sup., dense, irreg.; ye., rev. or.-bro. |
| CE39 | 1 | 1A | 1.10 ^eljkdighmf^ | 26.47 ^becd^ | aerial, dense, irreg.; or., rev. or.-red. | aerial, dense, irreg.; or., rev. or.-red. | sup., sparse, unif.; pale ye., rev. ye. | sup., dense, irreg.; ye., rev. or.-bro. |
| 63Ep | 1 | 1A | 1.05 ^ljknighm^ | 25.26 ^fecd^ | aerial, dense, irreg.; or., rev. or.-red. | aerial, dense, irreg.; or., rev. or.-red. | sup., sparse, unif.; pale ye., rev. ye. | sup., dense, irreg.; ye., rev. or.-bro. |
| CE9 | 1 | 1A | 1.03 ^oljknighmp^ | 24.72 ^gfecd^ | aerial, dense, irreg.; or., rev. or.-red. | aerial, dense, irreg.; or., rev. or.-red. | sup., sparse, unif.; pale ye., rev. ye. | sup., dense, irreg.; ye., rev. or.-bro. |
| 1F15 | 1 | 1A | 1.02 ^oljknihmp^ | 24.46 ^gfecd^ | aerial, dense, irreg.; or., rev. or.-red. | aerial, dense, irreg.; or., rev. or.-red. | sup., sparse, unif.; pale ye., rev. ye. | sup., dense, irreg.; ye., rev. or.-bro. |
| CE16 | 1 | 1A | 1.00 ^oljkniqhmp^ | 24.15 ^gfecd^ | aerial, dense, irreg.; or., rev. or.-red. | aerial, dense, irreg.; or., rev. or.-red. | sup., sparse, unif.; pale ye., rev. ye. | sup., dense, irreg.; ye., rev. or.-bro. |
| CE5 | 1 | 1A | 0.97 ^oljknqmp^ | 23.46 ^gfecdh^ | aerial, dense, irreg.; or., rev. or.-red. | aerial, dense, irreg.; or., rev. or.-red. | sup., sparse, unif.; pale ye., rev. ye. | sup., dense, irreg.; ye., rev. or.-bro. |
| CE18 | 1 | 1A | 0.97 ^oljknqmp^ | 23.43 ^gfecdh^ | aerial, dense, irreg.; or., rev. or.-red. | aerial, dense, irreg.; or., rev. or.-red. | sup., sparse, unif.; pale ye., rev. ye. | sup., dense, irreg.; ye., rev. or.-bro. |
| CE3 | 2 | ND | 0.96 ^olrknqmp^ | 23.13 ^gfecdh^ | sup., dense, irreg.; ye., rev. ye.-bro. | sup., dense, irreg.; ye., rev. ye.-bro. | sup., sparse, unif.; pale ye., rev. pale ye. | sup., dense, irreg.; ye., rev. ye.-bro. |
| P12 | 1 | 1A | 0.96 ^olrknqmp^ | 23.06 ^gfecdh^ | aerial, dense, irreg.; or., rev. or.-red. | aerial, dense, irreg.; or., rev. or.-red. | sup., sparse, unif.; pale ye., rev. ye. | sup., dense, irreg.; ye., rev. or.-bro. |
| 1F6 | 1 | ND | 0.94 ^olrnqmp^ | 22.70 ^gfeidh^ | aerial, dense, irreg.; or., rev. or.-red. | aerial, dense, irreg.; or., rev. or.-red. | sup., sparse, unif.; pale ye., rev. ye. | sup., dense, irreg.; ye., rev. or.-bro. |
| CE6 | 1 | 1A | 0.93 ^ornqsmp^ | 22.33 ^gfeidh^ | aerial, dense, irreg.; or., rev. or.-red. | aerial, dense, irreg.; or., rev. or.-red. | sup., sparse, unif.; pale ye., rev. ye. | sup., dense, irreg.; ye., rev. or.-bro. |
| P11 | 1 | 1B | 0.93 ^ornqsmp^ | 22.23 ^gfeidh^ | aerial, dense, irreg.; or., rev. or.-red. | aerial, dense, irreg.; or., rev. or.-red. | sup., dense, unif.; ye., rev. or.-red. | sup., dense, irreg.; ye., rev. or.-bro. |
| CE10 | 1 | 1A | 0.91 ^orntqsmp^ | 22.00 ^gfeidh^ | aerial, dense, irreg.; or., rev. or.-red. | aerial, dense, irreg.; or., rev. or.-red. | sup., sparse, unif.; pale ye., rev. ye. | sup., dense, irreg.; ye., rev. or.-bro. |
| CE2 | 1 | 1A | 0.87 ^orntqsup^ | 20.96 ^gfeijh^ | aerial, dense, irreg.; or., rev. or.-red. | aerial, dense, irreg.; or., rev. or.-red. | sup., sparse, unif.; pale ye., rev. ye. | sup., dense, irreg.; ye., rev. or.-bro. |
| CE22 | 1 | ND | 0.85 ^ortqsup^ | 20.45 ^gfkijh^ | aerial, dense, irreg.; or., rev. or.-red. | aerial, dense, irreg.; or., rev. or.-red. | sup., dense, unif.; ye., rev. or.-red. | sup., dense, irreg.; ye., rev. or.-bro. |
| CE7 | 1 | 1B | 0.82 ^wrvtqsu^ | 19.70 ^gfkiljh^ | aerial, dense, irreg.; or., rev. or.-red. | aerial, dense, irreg.; or., rev. or.-red. | sup., dense, unif.; ye., rev. or.-red. | sup., dense, irreg.; ye., rev. or.-bro. |
| 1F4 | 1 | 1B | 0.74 ^wyvtxsu^ | 17.75 ^nmkiljh^ | aerial, dense, irreg.; or., rev. or.-red. | aerial, dense, irreg.; or., rev. or.-red. | sup., dense, unif.; ye., rev. or.-red. | sup., dense, irreg.; ye., rev. or.-bro. |
| CE25 | 1 | 1A | 0.73 ^wyvtxu^ | 17.59 ^nmkiljh^ | aerial, dense, irreg.; or., rev. or.-red. | aerial, dense, irreg.; or., rev. or.-red. | sup., sparse, unif.; pale ye., rev. ye. | sup., dense, irreg.; ye., rev. or.-bro. |
| P98 | 1 | 1A | 0.71 ^wyvxu^ | 17.10 ^nmkiljo^ | aerial, dense, irreg.; or., rev. or.-red. | aerial, dense, irreg.; or., rev. or.-red. | sup., sparse, unif.; pale ye., rev. ye. | sup., dense, irreg.; ye., rev. or.-bro. |
| TH21Ep | 1 | 1B | 0.65^wyvxz^ | 15.66 ^nmkpljo^ | aerial, dense, irreg.; or., rev. or.-red. | aerial, dense, irreg.; or., rev. or.-red. | sup., dense, unif.; ye., rev. or.-red. | sup., dense, irreg.; ye., rev. or.-bro. |
| CE11 | 1 | 1B | 0.63 ^wyaxz^ | 15.30 ^nmkpljo^ | aerial, dense, irreg.; or., rev. or.-red. | aerial, dense, irreg.; or., rev. or.-red. | sup., dense, unif.; ye., rev. or.-red. | sup., dense, irreg.; ye., rev. or.-bro. |
| CE12 | 1 | 1B | 0.63 ^wyaxz^ | 15.13 ^nmkqpljo^ | aerial, dense, irreg.; or., rev. or.-red. | aerial, dense, irreg.; or., rev. or.-red. | sup., dense, unif.; ye., rev. or.-red. | sup., dense, irreg.; ye., rev. or.-bro. |
| P16 | 1 | 1B | 0.62 ^byaxz^ | 14.92 ^nmkqplro^ | aerial, dense, irreg.; or., rev. or.-red. | aerial, dense, irreg.; or., rev. or.-red. | sup., dense, unif.; ye., rev. or.-red. | sup., dense, irreg.; ye., rev. or.-bro. |
| CE24 | 1 | 1B | 0.60 ^byaxz^ | 14.40 ^nmsqplro^ | aerial, dense, irreg.; or., rev. or.-red. | aerial, dense, irreg.; or., rev. or.-red. | sup., dense, unif.; ye., rev. or.-red. | sup., dense, irreg.; ye., rev. or.-bro. |
| CE13 | 1 | 1B | 0.60 ^byaxz^ | 14.36 ^nmsqplro^ | aerial, dense, irreg.; or., rev. or.-red. | aerial, dense, irreg.; or., rev. or.-red. | sup., dense, unif.; ye., rev. or.-red. | sup., dense, irreg.; ye., rev. or.-bro. |
| CE27 | 1 | 1B | 0.55 ^byaz^ | 13.35 ^nmusqptro^ | aerial, dense, irreg.; or., rev. or.-red. | aerial, dense, irreg.; or., rev. or.-red. | sup., dense, unif.; ye., rev. or.-red. | sup., dense, irreg.; ye., rev. or.-bro. |
| CE29 | 1 | 1B | 0.55 ^byaz^ | 13.20 ^nusqptro^ | aerial, dense, irreg.; or., rev. or.-red. | aerial, dense, irreg.; or., rev. or.-red. | sup., dense, unif.; ye., rev. or.-red. | sup., dense, irreg.; ye., rev. or.-bro. |
| EpAr | 1 | 1B | 0.50 ^bacz^ | 12.10 ^nvusqptro^ | aerial, dense, irreg.; or., rev. or.-red. | aerial, dense, irreg.; or., rev. or.-red. | sup., dense, unif.; ye., rev. or.-red. | sup., dense, irreg.; ye., rev. or.-bro. |
| CBS 318.83 ***^e^*** | 1 | ND | 0.48 ^bacz^ | 11.61 ^wvusqptro^ | aerial, dense, irreg.; or., rev. or.-red. | aerial, dense, irreg.; or., rev. or.-red. | sup., sparse, unif.; pale ye.-ye. cl, rev. ye. | sup., dense, irreg.; ye., rev. or.-bro. |
| 79Ep | 1 | 1B | 0.44 ^bac^ | 10.73 ^wyvusqptrx^ | aerial, dense, irreg.; or., rev. or.-red. | aerial, dense, irreg.; or., rev. or.-red. | sup., dense, unif.; ye., rev. or.-red. | sup., dense, irreg.; ye., rev. or.-bro. |
| CE51 | 1 | ND | 0.43 ^bc^ | 10.42 ^zwyvusqptrx^ | aerial, dense, irreg.; or., rev. or.-red. | aerial, dense, irreg.; or., rev. or.-red. | sup., dense, unif.; ye., rev. or.-red. | sup., dense, irreg.; ye., rev. or.-bro. |

*^a^* Groups obtained by the phylogenetic analysis using the ITS1-5.8S-ITS2 region of the rDNA units, or by the ITS-RFLP analysis. *^b^* Groups and subgroups generated by AFLP analysis. *^c^* Means based on growth rate and lag phase duration in four different culture media, with three replicates (therefore, each number represents the mean value obtained from 12 independent measurements) . Means followed by the same letter in each column indicate that they were not statistically different (Tukey’s test, *P* > 5%). *^d^* Morphocultural characterization is presented with abbreviated words because of limited space in the table: sup., superficial, compact and homogeneous mycelium; unif., colony with uniform edge; irreg., colony with irregular edge; rev., reverse coloration of the colonies; or., orange; red., reddish; bro.,brownish; ye., yellowish; pale ye., pale yellowish. *^e^* Reference strains; CBS, Centraalbureau voor Schimmelcultures, Utrecht, Netherlands. (ND) Not determined.
