# Supplementary material for: Polyphasic Analysis of Intraspecific Diversity in Epicoccum nigrum Warrants Reclassification into Separate Species
Source: PLoS One. 2011 Aug 11;6(8):e14828. doi: 10.1371/journal.pone.0014828 (PMC3154903; doi:10.1371/journal.pone.0014828)
Supplement: Table S3 — Effect of culture media on mycelial growth and lag phase duration of 64 Epicoccum strains. (0.03 MB DOC) [file pone.0014828.s003.doc]

Table S3. Effect of culture media on mycelial growth and lag phase duration of 64 *Epicoccum* strains.

| **Culture media** | **Growth rate (cm.day^-1^) *^a^*** | **Lag phase (hours)*^a^*** |
| --- | --- | --- |
| Potato dextrose agar | 1.03 ^a^ | 14.62 ^a^ |
| Malt extract agar | 0.96 ^b^ | 14.86 ^a^ |
| Complete medium | 0.94 ^b^ | 12.53 ^b^ |
| Czapeck agar | 0.89 ^c^ | 14.98 ^a^ |

*^a^* Means followed by the same letter in each column indicate that they were not statistically different (Tukey’s test, *P* > 5%). Means based on growth rate and lag phase duration of 64 strains of *Epicoccum* in four different culture media, with three replicates (therefore, each number represents the mean value obtained from 192 independent measurements).
