# Supplementary material for: Polyphasic Analysis of Intraspecific Diversity in Epicoccum nigrum Warrants Reclassification into Separate Species
Source: PLoS One. 2011 Aug 11;6(8):e14828. doi: 10.1371/journal.pone.0014828 (PMC3154903; doi:10.1371/journal.pone.0014828)
Supplement: Table S4 — Conidial dimensions of 46 Epicoccum strains grown on PDA medium. (0.06 MB DOC) [file pone.0014828.s004.doc]

Table S4. Conidial dimensions of 46 *Epicoccum* strains grown on PDA medium.

| **Strain** | **ITS group *^a^*** | **AFLP group *^b^*** | **Length (μm)** | **Width (μm)** | **Length / Width ratio** |
| --- | --- | --- | --- | --- | --- |
| CE5 | 1 | 1A | 38.53 ^a^ | 28.20 ^ba^ | 1.37 ^ebdac^ |
| CE25 | 1 | 1A | 38.19 ^ba^ | 28.18 ^ba^ | 1.36 ^ebdac^ |
| CE16 | 1 | 1A | 38.14 ^ba^ | 28.05 ^ba^ | 1.36 ^ebdac^ |
| P17 | 1 | 1A | 37.75 ^ba^ | 27.71 ^ba^ | 1.36 ^ebdac^ |
| CE18 | 1 | 1A | 36.73 ^bc^ | 26.56 ^bdc^ | 1.38 ^bdac^ |
| CE9 | 1 | 1A | 35.37 ^dc^ | 25.96 ^edc^ | 1.36 ^ebdac^ |
| 1F15 | 1 | 1A | 34.43 ^de^ | 25.20 ^ed^ | 1.35 ^ebdac^ |
| CE29 | 1 | 1B | 38.66 ^a^ | 29.01 ^a^ | 1.34 ^ebdac^ |
| CE24 | 1 | 1B | 37.69 ^ba^ | 27.34 ^bac^ | 1.39 ^bdac^ |
| CE11 | 1 | 1B | 34.82 ^de^ | 24.70 ^e^ | 1.41 ^a^ |
| CE12 | 1 | 1B | 34.14 ^de^ | 25.07 ^ed^ | 1.36 ^ebdac^ |
| CE13 | 1 | 1B | 33.28 ^e^ | 24.31 ^e^ | 1.37 ^ebdac^ |
| CE27 | 1 | 1B | 30.58 ^f^ | 22.53 ^f^ | 1.35 ^ebdac^ |
| P16 | 1 | 1B | 25.51 ^hgi^ | 19.16 ^hgi^ | 1.33 ^ebdac^ |
| CE51 | 1 | ND | 35.55 ^dc^ | 25.98 ^edc^ | 1.37 ^ebdac^ |
| TC41 | 2 | 2A | 23.76 ^ji^ | 17.96 ^kmjli^ | 1.41 ^ba^ |
| C13B | 2 | 2A | 26.16 ^g^ | 19.08 ^hjgi^ | 1.37 ^ebdac^ |
| C41B | 2 | 2A | 25.35 ^hgi^ | 18.56 ^khjgi^ | 1.37 ^ebdac^ |
| SP2 | 2 | 2A | 22.64 ^kjl^ | 16.75 ^pmoln^ | 1.36 ^ebdac^ |
| TH1 | 2 | 2A | 25.04 ^hgi^ | 19.11 ^hjgi^ | 1.31 ^ed^ |
| TH2 | 2 | 2A | 24.36 ^hji^ | 18.19 ^khjli^ | 1.34 ^ebdac^ |
| C22B | 2 | 2B | 25.41 ^hgi^ | 19.00 ^hjgi^ | 1.32 ^edc^ |
| C41A | 2 | 2B | 25.07 ^hgi^ | 18.71 ^khjgi^ | 1.34 ^ebdac^ |
| P18 | 2 | 2B | 24.07 ^hji^ | 18.32 ^khjli^ | 1.30 ^e^ |
| CV2 | 2 | 2B | 17.67 ^r^ | 12.81 ^s^ | 1.38 ^bdac^ |
| Ep1sc | 2 | 2C | 21.96 ^kml^ | 15.86 ^poqn^ | 1.39 ^bac^ |
| P24 | 2 | ND | 26.69 ^g^ | 20.13 ^g^ | 1.33 ^ebdc^ |
| P13 | 2 | ND | 26.66 ^g^ | 19.86 ^hg^ | 1.34 ^ebdac^ |
| P31 | 2 | ND | 25.64 ^hg^ | 19.76 ^hg^ | 1.30 ^e^ |
| P64 | 2 | ND | 23.97 ^hji^ | 17.90 ^kmjli^ | 1.34 ^ebdac^ |
| P42 | 2 | ND | 23.81 ^ji^ | 18.56 ^khjgi^ | 1.29 ^e^ |
| P82 | 2 | ND | 23.13 ^kj^ | 17.46 ^kmjln^ | 1.32 ^edc^ |
| P910 | 2 | ND | 22.69 ^kjl^ | 17.07 ^kmoln^ | 1.33 ^ebdac^ |
| P83 | 2 | ND | 21.96 ^kml^ | 16.15 ^pon^ | 1.37 ^ebdac^ |
| P112 | 2 | ND | 21.82 ^kml^ | 16.33 ^pmon^ | 1.34 ^ebdac^ |
| P61 | 2 | ND | 21.33 ^nml^ | 15.68 ^proq^ | 1.36 ^ebdac^ |
| P44 | 2 | ND | 20.94 ^noml^ | 15.94 ^poqn^ | 1.31 ^edc^ |
| P62 | 2 | ND | 20.80 ^nomp^ | 15.39 ^proq^ | 1.35 ^ebdac^ |
| P21 | 2 | ND | 20.75 ^nomp^ | 15.73 ^proq^ | 1.32 ^edc^ |
| P58 | 2 | ND | 20.33 ^nomp^ | 15.21 ^prq^ | 1.32 ^edc^ |
| P91 | 2 | ND | 19.84 ^noqp^ | 15.37 ^prq^ | 1.29 ^e^ |
| P51 | 2 | ND | 19.45 ^oqp^ | 14.37 ^rqs^ | 1.36 ^ebdac^ |
| P81 | 2 | ND | 19.26 ^roqp^ | 14.32 ^rqs^ | 1.35 ^ebdac^ |
| P55 | 2 | ND | 19.08 ^rqp^ | 14.11 ^rs^ | 1.35 ^ebdac^ |
| P74 | 2 | ND | 19.08 ^rqp^ | 14.16 ^rs^ | 1.34 ^ebdac^ |
| P92 | 2 | ND | 18.43 ^rq^ | 14.09 ^rs^ | 1.31 ^ed^ |

*^a^* Groups obtained by phylogenetic or ITS-RFLP analysis using the ITS1-5.8S-ITS2 region of the rDNA units. *^b^* Groups and subgroups generated by AFLP analysis. Means (n = 85 conidia) followed by the same letter in each column indicate that they were not statistically different (Tukey’s test, *P* > 5%). (ND) Not determined.
