# Supplementary material for: Polyphasic Analysis of Intraspecific Diversity in Epicoccum nigrum Warrants Reclassification into Separate Species
Source: PLoS One. 2011 Aug 11;6(8):e14828. doi: 10.1371/journal.pone.0014828 (PMC3154903; doi:10.1371/journal.pone.0014828)
Supplement: Table S5 — Production of extracellular hydrolytic enzymes on solid media by 64 Epicoccum strains. (0.12 MB DOC) [file pone.0014828.s005.doc]

Table S5. Production of extracellular hydrolytic enzymes on solid media by 64 *Epicoccum* strains.

|  |  |  | **Production of hydrolytic enzymes on solid media *^c^*** | | | | | |
| --- | --- | --- | --- | --- | --- | --- | --- | --- |
| **Strain** | **ITS group *^a^*** | **AFLP group *^b^*** | **Polygalacturonase** | **Pectin lyase** | **Amylase** | **Lipase** | **Endoglucanase** | **Protease** |
| CBS 161.73 *^d^* | 2 | ND | 4.23 ^a^ | 4.83 ^a^ | 3.25 ^a^ | 3.44 ^a^ | ND | - |
| CBS 318.83 *^d^* | 1 | ND | 2.96 ^b^ | 3.97 ^b^ | 2.04 ^b^ | 1.55 ^oqnsmptlr^ | ND | - |
| CE25 | 1 | 1A | 2.05 ^c^ | 1.66 ^ioknhmflgj^ | 1.00 ^g^ | 2.53 ^becd^ | 1.00 ^p^ | - |
| CE51 | 1 | ND | 1.98 ^dc^ | 2.60 ^d^ | 1.32 ^dfce^ | 2.94 ^ba^ | 1.92 ^a^ | - |
| EpAr | 1 | 1B | 1.97 ^dce^ | 2.05 ^efg^ | 1.41 ^dc^ | 2.25 ^fhecidg^ | 1.27 ^nkmjl^ | - |
| CE24 | 1 | 1B | 1.84 ^gdfce^ | 2.10 ^ef^ | 1.17 ^dfge^ | 2.77 ^bc^ | 1.56 ^dce^ | - |
| CE29 | 1 | 1B | 1.83 ^gdfce^ | 2.07 ^efg^ | 1.22^dfge^ | 1.73 ^oqnsmpjkilr^ | 1.00 ^p^ | - |
| CE12 | 1 | 1B | 1.77 ^gdfceh^ | 1.67 ^iknhmflgj^ | 1.13 ^fge^ | 2.48 ^fbecd^ | 1.53 ^dfce^ | - |
| 79Ep | 1 | 1B | 1.68 ^gdfieh^ | 1.81 ^iehfgj^ | 1.53 ^c^ | 2.04 ^fhmejkilg^ | ND | - |
| CE13 | 1 | 1B | 1.66 ^gdfijeh^ | 2.62 ^d^ | 1.30 ^dfce^ | 2.22 ^fhejcidg^ | 1.80 ^a^ | - |
| TH21Ep | 1 | 1B | 1.62 ^gkfijeh^ | 3.34 ^c^ | 1.22 ^dfge^ | 1.98 ^ofnhmejkilg^ | 1.60 ^bc^ | - |
| CE7 | 1 | 1B | 1.60 ^gkfijlh^ | 1.27 ^ronmpq^ | 1.00 ^g^ | 1.45 ^oqnsptr^ | 1.00 ^p^ | - |
| 1F6 | 1 | ND | 1.53 ^gkmijlh^ | 1.52 ^iknhmolj^ | 1.16 ^dfge^ | 1.76 ^oqnhmpjkilr^ | 1.00 ^p^ | - |
| CE11 | 1 | 1B | 1.53 ^gkmijlh^ | 2.26 ^ed^ | 1.00 ^g^ | 1.67 ^oqnsmpjklr^ | 1.30 ^gkmjilh^ | - |
| CE27 | 1 | 1B | 1.52 ^gkmnijlh^ | 1.73 ^ikhflgj^ | 1.11 ^fg^ | 1.85 ^oqnhmpjkilg^ | 1.21 ^nmol^ | - |
| P16 | 1 | 1B | 1.43 ^okmnijlh^ | 1.74 ^ikhfgj^ | 1.00 ^g^ | 1.86 ^oqnhmpjkilg^ | 1.44 ^gdfcieh^ | - |
| P11 | 1 | 1B | 1.41 ^okmnijlh^ | 1.87 ^ehfg^ | 1.37 ^dce^ | 2.08 ^fhejkilg^ | 1.47 ^gdfce^ | - |
| CE22 | 1 | ND | 1.35 ^okmnijlp^ | 1.46 ^rioknhmplqj^ | 1.00 ^g^ | 2.04 ^fhmejkilg^ | 1.39 ^gkfjieh^ | - |
| CE10 | 1 | 1A | 1.32 ^okmnijlp^ | 1.56 ^ioknhmplqj^ | 1.00 ^g^ | 1.41 ^qsptr^ | 1.09 ^po^ | - |
| P98 | 1 | 1A | 1.30 ^okmnjlp^ | 1.62 ^ioknhmplgj^ | 1.37 ^dce^ | 1.56 ^oqnsmptlr^ | 1.27 ^nkmjil^ | - |
| P12 | 1 | 1A | 1.28 ^okmnlp^ | 1.32 ^roknmplq^ | 1.00 ^g^ | 2.48 ^fbecd^ | ND | - |
| 63Ep | 1 | 1A | 1.28 ^okmnlp^ | 1.72 ^ikhmflgj^ | 1.00 ^g^ | 2.41 ^fbecdg^ | 1.00 ^p^ | - |
| CE6 | 1 | 1A | 1.27 ^okmnlp^ | 1.20 ^ropq^ | 1.00 ^g^ | 1.56 ^oqnsmptlr^ | 1.23 ^nkmol^ | - |
| 1F15 | 1 | 1A | 1.27 ^okmnlp^ | 1.39 ^rioknmplqj^ | 1.00 ^g^ | 1.43 ^oqsptr^ | 1.19 ^nmo^ | - |
| P17 | 1 | 1A | 1.26 ^okmnlp^ | 1.26 ^ronmpq^ | 1.00 ^g^ | 2.08 ^fhejkilg^ | 1.11 ^npo^ | - |
| CE39 | 1 | 1A | 1.26 ^okmnlp^ | 1.33 ^roknmplq^ | 1.08 ^fg^ | 1.63 ^oqnsmpklr^ | 1.18 ^nmo^ | - |
| 1F4 | 1 | 1B | 1.25 ^omnlp^ | 1.40 ^rioknmplqj^ | 1.00 ^g^ | 2.03 ^fhmejkilg^ | 1.00 ^p^ | - |
| CE2 | 1 | 1A | 1.24 ^omnp^ | 1.20 ^ropq^ | 1.10 ^fg^ | 1.55 ^oqnsmptlr^ | 1.16 ^npmo^ | - |
| TH41Ep | 1 | ND | 1.22 ^omnp^ | 1.34 ^roknmplq^ | 1.17 ^dfge^ | 1.48 ^oqnsmptr^ | 1.28 ^nkmjil^ | - |
| CE9 | 1 | 1A | 1.19 ^omnp^ | 1.18 ^rpq^ | 1.00 ^g^ | 1.67 ^oqnsmpjklr^ | 1.28 ^nkmjil^ | - |
| CE18 | 1 | 1A | 1.16 ^onp^ | 1.66 ^ioknhmflgj^ | 1.00 ^g^ | 2.30 ^fhecdg^ | 1.23 ^nkmol^ | - |
| CE5 | 1 | 1A | 1.16 ^onp^ | 1.00 ^r^ | 1.00 ^g^ | 1.61 ^oqnsmplr^ | 1.00 ^p^ | - |
| C33Ep | 1 | 1A | 1.16 ^onp^ | 1.84 ^iehfg^ | 1.38 ^dce^ | 1.94 ^ofnhmpjkilg^ | 1.59 ^c^ | - |
| CE16 | 1 | 1A | 1.14 ^op^ | 1.20 ^ropq^ | 1.00 ^g^ | 1.40 ^qsptr^ | 1.15 ^npmo^ | - |
| CE3 | 2 | ND | 1.00 ^p^ | 1.00 ^r^ | 1.00 ^g^ | 1.00 ^t^ | 1.00 ^p^ | - |
| CV1 | 2 | ND | 1.93 ^dfce^ | 1.49 ^ioknhmplqj^ | 1.27 ^dfe^ | 2.68 ^bcd^ | 1.76 ^ba^ | - |
| Ep1sc | 2 | 2C | 1.35 ^okmnijlp^ | 1.42 ^rioknhmplqj^ | 1.00 ^g^ | 2.20 ^fhejkidg^ | 1.00 ^p^ | - |
| C42A | 2 | 2A | 1.25 ^omnlp^ | 1.37 ^roknmplqj^ | 1.08 ^fg^ | 2.02 ^fnhmejkilg^ | ND | - |
| C22B | 2 | 2B | 1.23 ^omnp^ | 1.25 ^ronpq^ | 1.00 ^g^ | 1.00 ^t^ | 1.46 ^gdfce^ | - |
| P18 | 2 | 2B | 1.20 ^omnp^ | 1.00 ^r^ | 1.00 ^g^ | 1.00 ^t^ | 1.45 ^gdfceh^ | - |
| 62Ep | 2 | 2A | 1.00 ^p^ | 1.00 ^r^ | 1.00 ^g^ | 1.27 ^str^ | 1.00 ^p^ | - |
| C12B | 2 | ND | 1.00 ^p^ | 1.20 ^ropq^ | 1.00 ^g^ | 1.00 ^t^ | 1.28 ^nkmjilh^ | - |
| C12C | 2 | ND | 1.00 ^p^ | 1.24 ^ronpq^ | 1.00 ^g^ | 1.43 ^oqsptr^ | 1.53 ^dfce^ | - |
| C13B | 2 | 2A | 1.00 ^p^ | 1.12 ^rq^ | 1.00 ^g^ | 1.16 ^st^ | 1.00 ^p^ | - |
| CV2 | 2 | 2B | 1.00 ^p^ | 1.22 ^ronpq^ | 1.00 ^g^ | 1.00 ^t^ | 1.00 ^p^ | - |
| P13 | 2 | ND | 1.00 ^p^ | 1.00 ^r^ | 1.00 ^g^ | 1.26 ^str^ | 1.57 ^dc^ | - |
| C13A | 2 | 2A | 1.00 ^p^ | 1.10 ^rq^ | 1.00 ^g^ | 1.16 ^st^ | 1.00 ^p^ | - |
| C12A | 2 | 2A | 1.00 ^p^ | 1.00 ^r^ | 1.00 ^g^ | 1.50 ^oqnsmptr^ | 1.15 ^npmo^ | - |
| C41B | 2 | 2A | 1.00 ^p^ | 1.24 ^ronpq^ | 1.00 ^g^ | 1.16 ^st^ | 1.00 ^p^ | - |
| CV3 | 2 | 2B | 1.00 ^p^ | 1.12 ^rq^ | 1.00 ^g^ | 1.00 ^t^ | 1.00 ^p^ | - |
| SP1 | 2 | 2A | 1.00 ^p^ | 1.00 ^r^ | 1.00 ^g^ | 1.53 ^oqnsmptlr^ | 1.00 ^p^ | - |
| SP2 | 2 | 2A | 1.00 ^p^ | 1.00 ^r^ | 1.00 ^g^ | 1.36 ^qstr^ | 1.00 ^p^ | - |
| TC2 | 2 | ND | 1.00 ^p^ | 1.18 ^rpq^ | 1.00 ^g^ | 1.00 ^t^ | 1.00 ^p^ | - |
| TC1 | 2 | 2A | 1.00 ^p^ | 1.14 ^rq^ | 1.00 ^g^ | 1.22 ^str^ | 1.00 ^p^ | - |
| TC42F | 2 | 2A | 1.00 ^p^ | 1.13 ^rq^ | 1.00 ^g^ | 1.00 ^t^ | 1.19 ^nmo^ | - |
| TC41 | 2 | 2A | 1.00 ^p^ | 1.00 ^r^ | 1.00 ^g^ | 1.55 ^oqnsmptlr^ | 1.00 ^p^ | - |
| TC42A | 2 | 2A | 1.00 ^p^ | 1.00 ^r^ | 1.00 ^g^ | 1.28 ^str^ | 1.41 ^gdfjieh^ | - |
| TH1 | 2 | 2A | 1.00 ^p^ | 1.26 ^ronmpq^ | 1.00 ^g^ | 1.17 ^st^ | 1.00 ^p^ | - |
| TH13F | 2 | ND | 1.00 ^p^ | 1.28 ^ronmplq^ | 1.00 ^g^ | 1.59 ^oqnsmplr^ | 1.39 ^gkfjieh^ | - |
| TH2 | 2 | 2A | 1.00 ^p^ | 1.00 ^r^ | 1.00 ^g^ | 1.20 ^str^ | 1.00 ^p^ | - |
| C22A | 2 | ND | 1.00 ^p^ | 1.27 ^ronmpq^ | 1.00 ^g^ | 1.24 ^str^ | 1.00 ^p^ | - |
| TH31A | 2 | ND | 1.00 ^p^ | 1.00 ^r^ | 1.00 ^g^ | 1.42 ^oqsptr^ | 1.00 ^p^ | - |
| TH31B | 2 | 2A | 1.00 ^p^ | 1.25 ^ronpq^ | 1.00 ^g^ | 1.72 ^oqnsmpjkilr^ | 1.00 ^p^ | - |
| C41A | 2 | 2B | 1.00 ^p^ | 1.00 ^r^ | 1.00 ^g^ | 1.00 ^t^ | 1.36 ^gkfjilh^ | - |

*^a^* Groups obtained by the phylogenetic analysis using the ITS1-5.8S-ITS2 region of the rDNA units, or by the ITS-RFLP analysis. *^b^* Groups and subgroups generated by AFLP analysis. *^c^* Results are the mean of three independent experiments and represent the enzyme activities expressed as the ratio between the diameters of the degraded zone and the colony. Values >1 indicate enzyme secretion into the medium resulting in a degraded zone around the colony. Values =1 indicate degraded zones only under the colony. Means followed by the same letter in each column indicate that they were not statistically different (Tukey’s test, *P* > 5%). *^d^* Reference strains; CBS, Centraalbureau voor Schimmelcultures, Utrecht, Netherlands. (ND) Not determined. (-) Indicates no degraded zone or that it could not be estimated.
