# Supplementary material for: Polyphasic Analysis of Intraspecific Diversity in Epicoccum nigrum Warrants Reclassification into Separate Species
Source: PLoS One. 2011 Aug 11;6(8):e14828. doi: 10.1371/journal.pone.0014828 (PMC3154903; doi:10.1371/journal.pone.0014828)
Supplement: Table S6 — Phenotypic characterization of Epicoccum chlorate-resistant mutants. (0.11 MB DOC) [file pone.0014828.s006.doc]

Table S6. Phenotypic characterization^a^ of *Epicoccum* chlorate-resistant mutants.

|  |  |  | **Nº of mutants in each phenotypic class** | | | |
| --- | --- | --- | --- | --- | --- | --- |
| **Strains** | **Obtained mutants** | **Characterized mutants** | ***nit1*** | ***nit3*** | **NitM** | ***crn*** |
| TH31B | 3 | 2 | 1 | - | - | 1 |
| TC2 | 5 | 1 | 1 | - | - | - |
| TH1 | 4 | 3 | 1 | - | - | 2 |
| TH13F | 3 | 2 | 1 | - | - | 1 |
| C41B | 3 | 3 | 2 | - | 1 | - |
| P21 | 3 | 3 | 2 | - | - | 1 |
| P24 | 2 | 1 | 1 | - | - | - |
| CE3 | 6 | 3 | 1 | 2 | - | - |
| P11 | 4 | 2 | 1 | 1 | - | - |
| CE6 | 5 | 2 | 1 | - | - | 1 |
| CE7 | 5 | 2 | 2 | - | - | - |
| CE10 | 3 | 2 | 2 | - | - | - |
| CE11 | 3 | 2 | 2 | - | - | - |
| CE12 | 4 | 2 | - | 2 | - | - |
| CE13 | 4 | 2 | - | 1 | - | 1 |
| CE24 | 4 | 1 | 1 | - | - | - |
| CE27 | 4 | 1 | - | - | 1 | - |
| CE29 | 4 | 2 | - | 1 | - | 1 |
| P12 | 3 | 1 | 1 | - | - | - |
| P16 | 5 | 1 | - | - | 1 | - |
| P17 | 4 | 2 | 1 | - | - | 1 |
| 79Ep | 5 | 2 | 1 | - | - | 1 |
| SP1 | 7 | 1 | - | - | - | 1 |
| C42A | 4 | 2 | - | - | - | 2 |
| TC41F | 3 | 1 | - | - | - | 1 |
| P31 | 5 | 1 | - | - | - | 1 |
| P32 | 5 | 2 | - | - | - | 2 |
| P33 | 8 | 2 | - | - | - | 2 |
| P44 | 3 | 1 | - | - | - | 1 |
| P112 | 5 | 1 | - | - | - | 1 |
| P81 | 2 | 1 | - | - | - | 1 |
| CE25 | 2 | 1 | - | - | - | 1 |
| CE39 | 2 | 1 | - | - | - | 1 |
| P98 | 4 | 1 | - | - | - | 1 |
| C13A | 2 | NC |  |  |  |  |
| C13B | 2 | NC |  |  |  |  |
| TH31A | 1 | NC |  |  |  |  |
| TC1 | 1 | NC |  |  |  |  |
| CV2 | 1 | NC |  |  |  |  |
| TH2 | 3 | NC |  |  |  |  |
| SP2 | 3 | NC |  |  |  |  |
| 62Ep | 2 | NC |  |  |  |  |
| Ep1sc | 2 | NC |  |  |  |  |
| TC42A | 2 | NC |  |  |  |  |
| C22A | 1 | NC |  |  |  |  |
| C22B | 2 | NC |  |  |  |  |
| C41A | 3 | NC |  |  |  |  |
| C12A | 3 | NC |  |  |  |  |
| C12C | 2 | NC |  |  |  |  |
| P13 | 10 | NC |  |  |  |  |
| P18 | 3 | NC |  |  |  |  |
| P311 | 4 | NC |  |  |  |  |
| P42 | 3 | NC |  |  |  |  |
| P102 | 3 | NC |  |  |  |  |
| P104 | 4 | NC |  |  |  |  |
| P52 | 2 | NC |  |  |  |  |
| P57 | 2 | NC |  |  |  |  |
| P58 | 3 | NC |  |  |  |  |
| P61 | 4 | NC |  |  |  |  |
| P62 | 3 | NC |  |  |  |  |
| P64 | 4 | NC |  |  |  |  |
| P71 | 2 | NC |  |  |  |  |
| P83 | 2 | NC |  |  |  |  |
| P96 | 3 | NC |  |  |  |  |
| P97 | 2 | NC |  |  |  |  |
| P911 | 2 | NC |  |  |  |  |
| P912 | 4 | NC |  |  |  |  |
| CE2 | 4 | NC |  |  |  |  |
| CE5 | 3 | NC |  |  |  |  |
| CE51 | 12 | NC |  |  |  |  |
| CE9 | 6 | NC |  |  |  |  |
| CE16 | 2 | NC |  |  |  |  |
| CE18 | 1 | NC |  |  |  |  |
| CE22 | 1 | NC |  |  |  |  |
| EpAr | 5 | NC |  |  |  |  |
| 63Ep | 3 | NC |  |  |  |  |
| TH41Ep | 4 | NC |  |  |  |  |
| TH21Ep | 4 | NC |  |  |  |  |

^a^ Chlorate-resistant mutants phenotype was determined by growth tests in culture medium with one of several nitrogen sources (nitrate, nitrite, hypoxanthine, glutamate). *nit1*, mutation in the nitrate reductase structural locus; *nit3*, mutation in the pathway-specific regulatory locus; NitM, mutations in the molybdenum cofactor loci; *crn*, mutation in the permease locus. (NC) Not characterized.
