# Supplementary material for: Polyphasic Analysis of Intraspecific Diversity in Epicoccum nigrum Warrants Reclassification into Separate Species
Source: PLoS One. 2011 Aug 11;6(8):e14828. doi: 10.1371/journal.pone.0014828 (PMC3154903; doi:10.1371/journal.pone.0014828)
Supplement: Table S7 — GenBank accession numbers of ITS1-5.8S-ITS2 and β-tubulin sequences used in this study. (0.10 MB DOC) [file pone.0014828.s007.doc]

Table S7. GenBank accession numbers of ITS1-5.8S-ITS2 and β-tubulin sequences used in this study.

|  | **GenBank accession number** | |  |  |
| --- | --- | --- | --- | --- |
| **Species** | **ITS1-5.8S-ITS2** | **β-tubulin** | **Host / Source** | **Country** |
| *Phoma* *herbarum* CBS 502.91 | GU237874 | GU237613 | *Nerium* sp. | Netherlands |
| *Phoma* *herbarum* CBS 615.75 | FJ427022 | FJ427133 | *Rosa multiflora* | Netherlands |
| *Phoma* *glomerata* CBS 464.97 | FJ427012 | FJ427123 | Indoor | Netherlands |
| *Phoma* *glomerata* CBS 528.66 | FJ427013 | FJ427124 | *Chrysanthemum* sp. | Netherlands |
| *Phoma* *eupyrena* CBS 374.91 | FJ426999 | FJ427110 | *Solanum tuberosum* | Netherlands |
| *Phoma* *eupyrena* CBS 527.66 | FJ427000 | FJ427111 | Wheat field soil | Germany |
| *Phoma huancayensis* CBS 105.80 | GU237732 | GU237615 | Solanum sp. | Peru |
| *Phoma huancayensis* CBS 390.93 | GU237857 | GU237616 | Chenopodium quinoa | Peru |
| *Phoma exigua* var. *exigua* CBS 431.74 | FJ427001 | FJ427112 | *Solanum tuberosum* | Netherlands |
| *Phoma zantedeschiae* CBS 131.93 | FJ427084 | FJ427188 | *Calla* sp. | Netherlands |
| *Phoma pimprina* CBS 246.60 ^a^ | FJ427049 | FJ427159 | Soil | India |
| *Phoma pimprina* PD 77/1028 ^a^ | FJ427050 | FJ427160 | Soil | India |
| *Phoma sorghina* CBS 179.80 ^b^ | FJ427067 | FJ427173 | *Sorghum vulgare* | Puerto Rico |
| *Phoma sorghina* CBS 180.80 ^b^ | FJ427068 | FJ427174 | *Zea mays* | South Africa |
| *Phoma sorghina* CBS 181.80 ^b^ | FJ427069 | FJ427175 | *Oryza sativa* | Guinea-Bissau |
| *Phoma sorghina* CBS 293.72 ^b^ | FJ427070 | FJ427176 | *Panicum miliare* | India |
| *Phoma sorghina* CBS 301.89 ^b^ | FJ427071 | FJ427177 | *Lycopersicon esculentum* | Martinique |
| *Phoma sorghina* CBS 627.68 ^b^ | FJ427072 | FJ427178 | *Citrus* sp. | France |
| *Phoma sorghina* CBS 846.68 ^b^ | FJ427073 | FJ427179 | *Coffea* sp. | India |
| *Phoma sorghina* CBS 886.95 ^b^ | FJ427074 | FJ427180 | *Stellaria* sp. | Papua New Guinea |
| *Phoma sorghina* CBS 986.95 ^b^ | FJ427075 | FJ427181 | Soil | Papua New Guinea |
| *Phoma sorghina* PD 76/1025 ^b^ | FJ427076 | FJ427182 | *Aspidiotus destructor* | India |
| *Phoma sorghina* PD 81/721 ^b^ | FJ427077 | FJ427183 | *Pinus* sp. | USA |
| *Phoma sorghina* PD 88/549 ^b^ | FJ427078 | FJ427184 | *Lycopersicon esculentum* | Martinique |
| *Phoma sorghina* PD 03486771 ^b^ | FJ427079 | FJ427185 | *Triticum* sp. | Netherlands |
| *Phoma epicoccina* CBS 125.82 ^c^ | FJ426995 | FJ427106 | Toe nail (human) | Netherlands |
| *Phoma epicoccina* CBS 173.73 ^c^ | FJ426996 | FJ427107 | Seeds of *Dactylis glomerata* | USA |
| *Phoma epicoccina* CBS 505.85 ^c^ | FJ426997 | FJ427108 | Soil | Germany |
| *Phoma epicoccina* CBS 115825 ^c^ | FJ426998 | FJ427109 | *Malus* sp. | Netherlands |
| *Phoma epicoccina* ^c^ | AF149931 | - | Seedlings of *Picea sitchensis* | United Kingdon |
| *Phoma epicoccina* ^c^ | AF149932 | - | Seedlings of *Picea sitchensis* | United Kingdon |
| *Phoma epicoccina* ^c^ | AF149933 | - | PVA emulsion | United Kingdon |
| *Phoma epicoccina* ^c^ | AF149934 | - | Toe nail (human) | Netherlands |
| *Epicoccum andropogonis* | AJ306620 | - | *Claviceps pusilla* on *Heteropogon contortus* | Zimbabwe |
| *Epicoccum andropogonis* | AJ400905 | - | *Claviceps purpurea* on *Festuca arundinacea* | Czech Republic |
| ***Epicoccum nigrum* ATCC 32948** | FJ424240 | - | Jet fuel | New Zealand |
| *Epicoccum nigrum* ATCC 62191 | FJ424241 | - | *Heterodera glycines* cyst | USA |
| *Epicoccum nigrum* ATCC 96794 | FJ424258 | - | *Prunus persica* twigs | Spain |
| *Epicoccum nigrum* | AF149926 | - | Cortex of *Vismia* sp. | Colombia |
| *Epicoccum nigrum* | AF149927 | - | *Resupinatus applicatus* on *Salix* sp. | Spain |
| *Epicoccum nigrum* | AF149928 | - | Branches of *Thymus mastichina* | Spain |
| *Epicoccum nigrum* | AF149929 | - | - | Ecuador |
| *Epicoccum nigrum* | AF149930 | - | - | Ecuador |
| *Epicoccum nigrum* | AF455395 | - | Nasal mucosa (human) | Austria |
| *Epicoccum nigrum* | AF455403 | - | Nasal mucosa (human) | Austria |
| *Epicoccum nigrum* | AF455409 | - | Nasal mucosa (human) | Austria |
| *Epicoccum nigrum* | AF455447 | - | Nasal mucosa (human) | Austria |
| *Epicoccum nigrum* | AF455455 | - | Nasal mucosa (human) | Austria |
| *Epicoccum nigrum* | FJ424232 | - | *Prunus persica* twigs | Spain |
| *Epicoccum nigrum* | FJ424233 | - | *Prunus persica* twigs | Spain |
| *Epicoccum nigrum* | FJ424234 | - | *Prunus persica* twigs | Spain |
| *Epicoccum nigrum* | FJ424235 | - | *Prunus persica* twigs | Spain |
| *Epicoccum nigrum* | FJ424236 | - | *Prunus persica* twigs | Spain |
| *Epicoccum nigrum* | FJ424237 | - | *Prunus persica* twigs | Spain |
| *Epicoccum nigrum* | FJ424238 | - | *Prunus persica* twigs | Spain |
| *Epicoccum nigrum* | FJ424239 | - | *Prunus persica* flowers | Spain |
| *Epicoccum nigrum* | FJ424242 | - | *Quercus* sp. | Italy |
| *Epicoccum nigrum* | FJ424243 | - | *Prunus persica* twigs | Spain |
| *Epicoccum nigrum* | FJ424244 | - | *Prunus persica* twigs | Spain |
| *Epicoccum nigrum* | FJ424245 | - | *Prunus persica* twigs | Spain |
| *Epicoccum nigrum* | FJ424246 | - | *Prunus persica* flowers | Spain |
| *Epicoccum nigrum* | FJ424247 | - | *Prunus persica* flowers | Spain |
| *Epicoccum nigrum* | FJ424248 | - | *Prunus persica* flowers | Spain |
| *Epicoccum nigrum* | FJ424249 | - | *Prunus persica* flowers | Spain |
| *Epicoccum nigrum* | FJ424250 | - | *Prunus persica* flowers | Spain |
| *Epicoccum nigrum* | FJ424251 | - | *Prunus persica* flowers | Spain |
| *Epicoccum nigrum* | FJ424252 | - | *Prunus persica* flowers | Spain |
| *Epicoccum nigrum* | FJ424253 | - | *Prunus persica* flowers | Spain |
| *Epicoccum nigrum* | FJ424254 | - | *Prunus persica* flowers | Spain |
| *Epicoccum nigrum* | FJ424255 | - | *Prunus persica* flowers | Spain |
| *Epicoccum nigrum* | FJ424256 | - | *Prunus persica* flowers | Spain |
| *Epicoccum nigrum* | FJ424257 | - | *Prunus persica* flowers | Spain |
| *Epicoccum nigrum* | FJ424259 | - | *Prunus persica* twigs | Spain |
| *Epicoccum nigrum* | FJ424260 | - | *Prunus persica* flowers | Spain |
| *Epicoccum nigrum* | FJ424261 | - | *Prunus persica* flowers | Spain |
| *Epicoccum nigrum* | FJ424262 | - | *Prunus persica* flowers | Spain |
| *Epicoccum nigrum* | FJ424263 | - | *Prunus persica* flowers | Spain |
| *Epicoccum nigrum* | FJ424264 | - | *Prunus persica* twigs | Spain |
| *Epicoccum nigrum* | EU529998 | - | Endophytic of *Vitis vinifera* | Italy |
| *Epicoccum nigrum* | EU530001 | - | Endophytic of *Vitis vinifera* | Italy |
| *Epicoccum nigrum* | AY521445 | - | Endophytic of *Pinus* spp. | China |
| *Epicoccum nigrum* | AY521446 | - | Endophytic of *Pinus* spp. | China |
| *Epicoccum nigrum* | AY521447 | - | Endophytic of *Pinus* spp. | China |
| *Epicoccum nigrum* | AY521448 | - | Endophytic of *Pinus* spp. | China |
| *Epicoccum nigrum* | AY521449 | - | Endophytic of *Pinus* spp. | China |
| *Epicoccum nigrum* | AY521450 | - | Endophytic of *Pinus* spp. | China |
| *Epicoccum nigrum* | AY341003 | - | Endophytic of *Pinus* spp. | China |
| *Epicoccum nigrum* | AY341002 | - | Endophytic of *Pinus* spp. | China |
| *Epicoccum nigrum* | AY341001 | - | Endophytic of *Pinus* spp. | China |
| *Epicoccum nigrum* | AY787697 | - | Xylem of *Fraxinus excelsior* | Lithuania |
| *Epicoccum nigrum* | DQ093668 | - | *Pinus sylvestris* decayed root | Lithuania |
| *Epicoccum nigrum* | AY618231 | - | Xylem of *Picea abies* | Sweden |
| *Epicoccum nigrum* | FJ228164 | - | *Fraxinus excelsior* | Sweden |
| *Epicoccum nigrum* | AY625064 | - | Dust samples from residential homes | USA |
| *Epicoccum nigrum* | AY093413 | - | Culture of *Xanthophyllomyces dendrorhous* | USA |
| *Epicoccum nigrum* | DQ026007 | - | Seeds of *Amaranthus hypochondriacus* | Mexico |
| *Epicoccum nigrum* | FN548154 | - | Leaves of *Fagus sylvatica* | Germany |
| *Epicoccum nigrum* | AY160213 | - | *Pinus nigra* | USA |
| *Epicoccum nigrum* | FJ791156 | - | Old parchment document | Portugal |
| *Epicoccum nigrum* | AY391835 | - | Lignite | Slovak Republic |
| *Epicoccum nigrum* | FJ605251 | - | Asymptomatic root of *Maxillaria rigida* | Brazil |
| *Epicoccum* sp. | AY305359 | - | *Humulus lupulus* | France |
| *Epicoccum* sp. | AY305360 | - | *Humulus lupulus* | France |
| *Epicoccum* sp. | AY305361 | - | *Humulus lupulus* | France |
| *Epicoccum* sp. | AY305362 | - | *Humulus lupulus* | France |
| *Epicoccum* sp. | AY305363 | - | *Humulus lupulus* | France |
| *Epicoccum* sp. | AY305364 | - | *Humulus lupulus* | France |
| *Epicoccum* sp. | AJ279452 | - | Endophytic of *Phragmites australis* | Germany |
| *Epicoccum* sp. | AJ279463 | - | Endophytic of *Phragmites australis* | Germany |
| *Epicoccum* sp. | AJ279486 | - | Endophytic of *Phragmites australis* | Germany |
| *Epicoccum* sp. | FJ210548 | - | Kernel of *Zea mays* | USA |
| *Epicoccum* sp. | FJ210549 | - | Stalk of *Zea mays* | USA |
| *Epicoccum* sp. | FJ210550 | - | Kernel of *Zea mays* | USA |
| *Epicoccum* sp. | FJ210551 | - | Leaves of *Zea mays* | USA |
| *Epicoccum* sp. | FJ210552 | - | Stalk of *Zea mays* | USA |
| *Epicoccum* sp. | FJ210553 | - | Stalk of *Zea mays* | USA |
| *Epicoccum* sp. | FJ210554 | - | Kernel of *Zea mays* | USA |
| *Epicoccum* sp. | FJ210555 | - | Leaves of *Zea mays* | USA |
| *Epicoccum* sp. | EF589869 | - | Endophytic of *Centaurea stoebe* | USA |
| *Epicoccum* sp. | FJ176473 | - | Endophytic of *Acer ginnala* | China |

^a^ Synonym = *Epicoccum pimprinum*. ^b^ Synonym = *Epicoccum sorghi*. ^c^ Synonym = *Epicoccum nigrum*. ATCC (American Type Culture Collection). CBS (Centraalbureau voor Schimmelcultures, Utrecht, Netherlands).
