# Supplementary figures and images for: Polyphasic Analysis of Intraspecific Diversity in Epicoccum nigrum Warrants Reclassification into Separate Species
Source: PLoS One. 2011 Aug 11;6(8):e14828. doi: 10.1371/journal.pone.0014828 (PMC3154903; doi:10.1371/journal.pone.0014828)

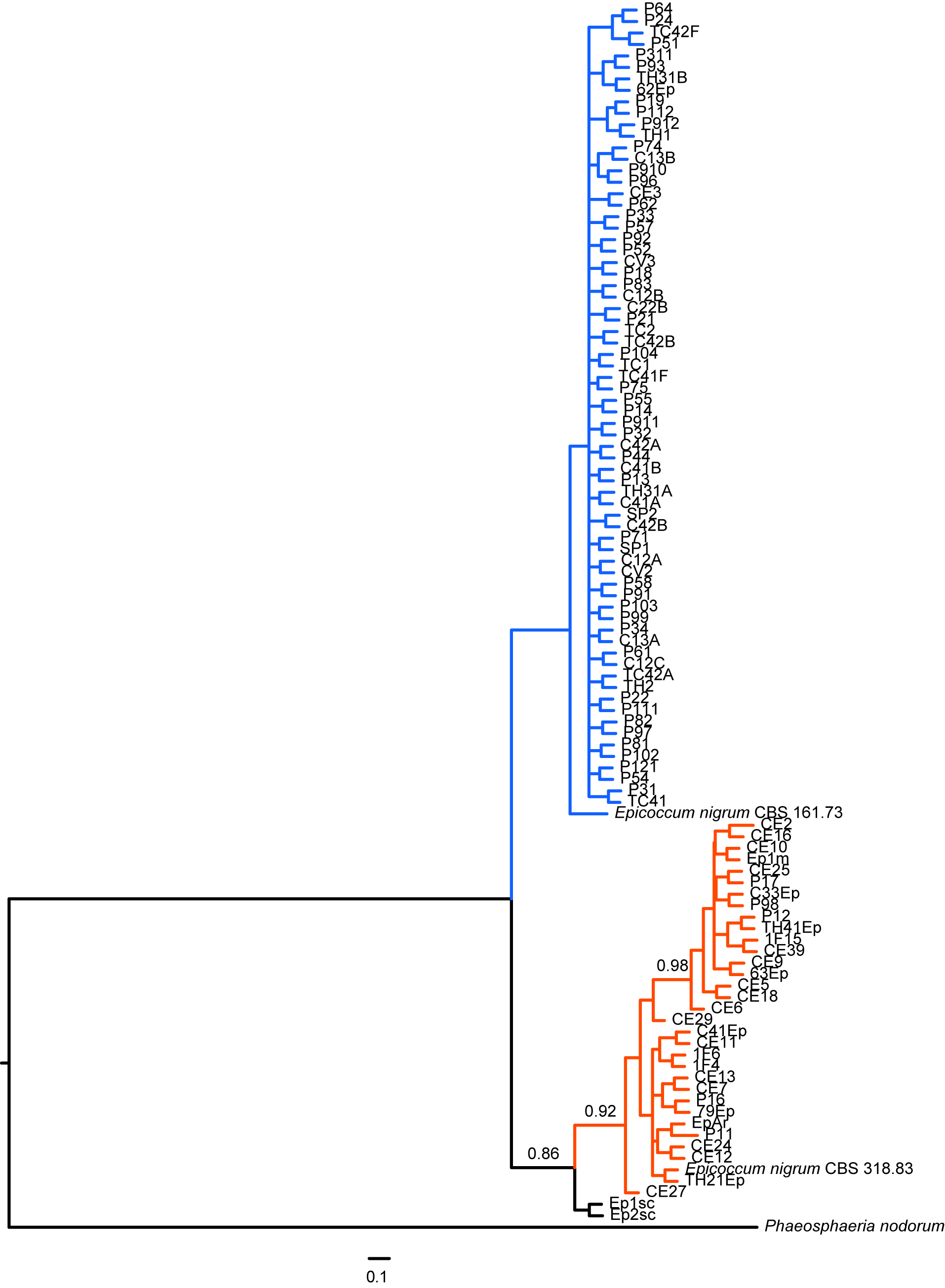

Supplement: Figure S1 — Bayesian maximum clade credibility tree inferred using ITS1-5.8S-ITS2 sequences of 106 Epicoccum strains. Two E. nigrum reference strains (CBS 318.83 and CBS 161.73) were included. P. nodorum access AF250830 was used as outgroup. The posterior probability values are shown next to relevant nodes. Orange lines represent the Epicoccum strains from group 1. Blue and black lines represent the strains from group 2. (0.56 MB TIF ) [file pone.0014828.s008.tif]

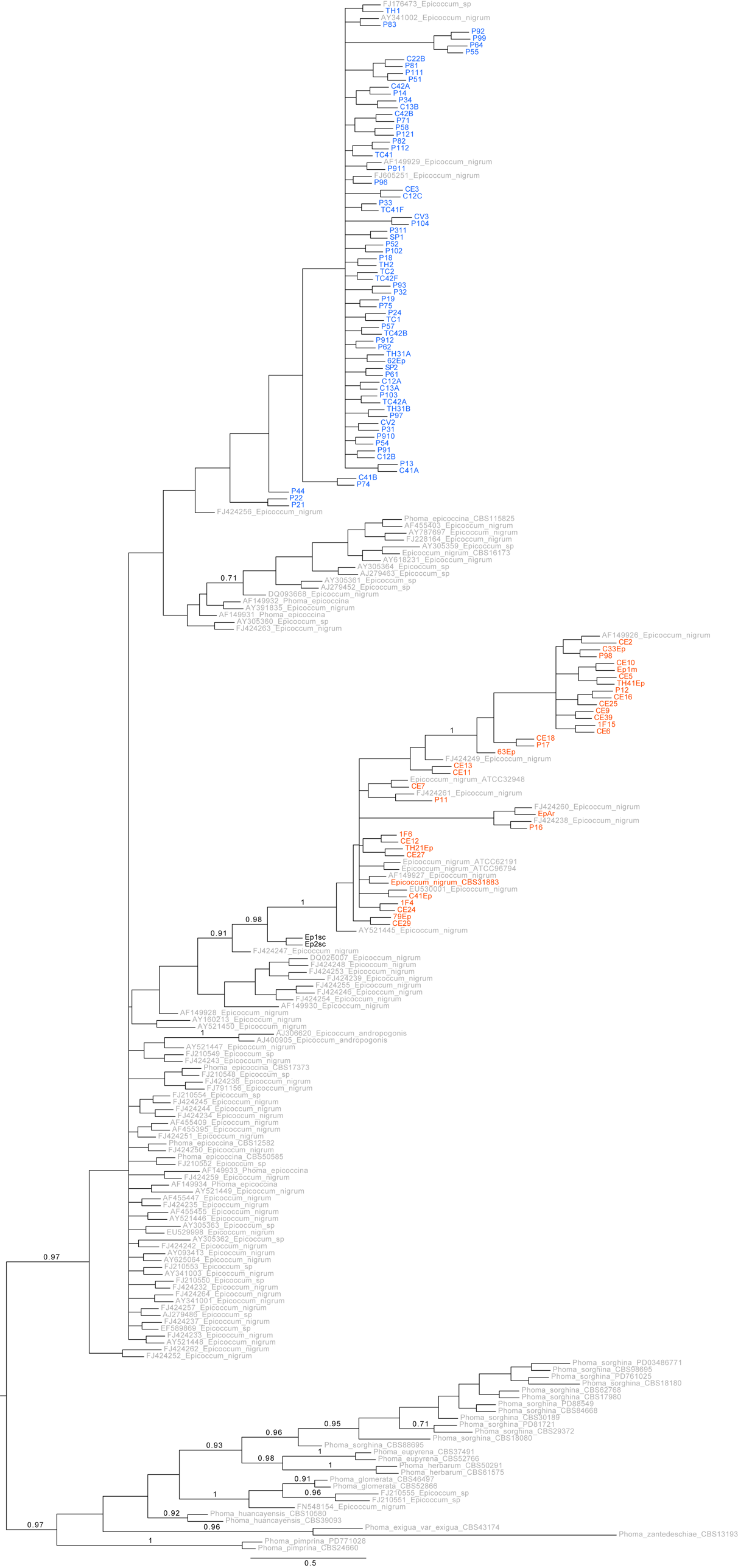

Supplement: Figure S2 — Bayesian maximum clade credibility tree inferred using ITS1-5.8S-ITS2 sequences of 226 taxa, including 106 Epicoccum strains in the present study and other accessions of Epicoccum and Phoma from GenBank. The ITS gene sequences of P. zantedeschiae CBS 131.93 and P. exigua var. exigua CBS 431.74 were used as outgroups. The posterior probability values are shown next to relevant nodes. Strains from group 1 in this study are indicated in orange color, and strains from group 2 in this study are indicated in blue and black colors. Accessions from GenBank are typed in gray. (3.24 MB TIF) [file pone.0014828.s009.tif]

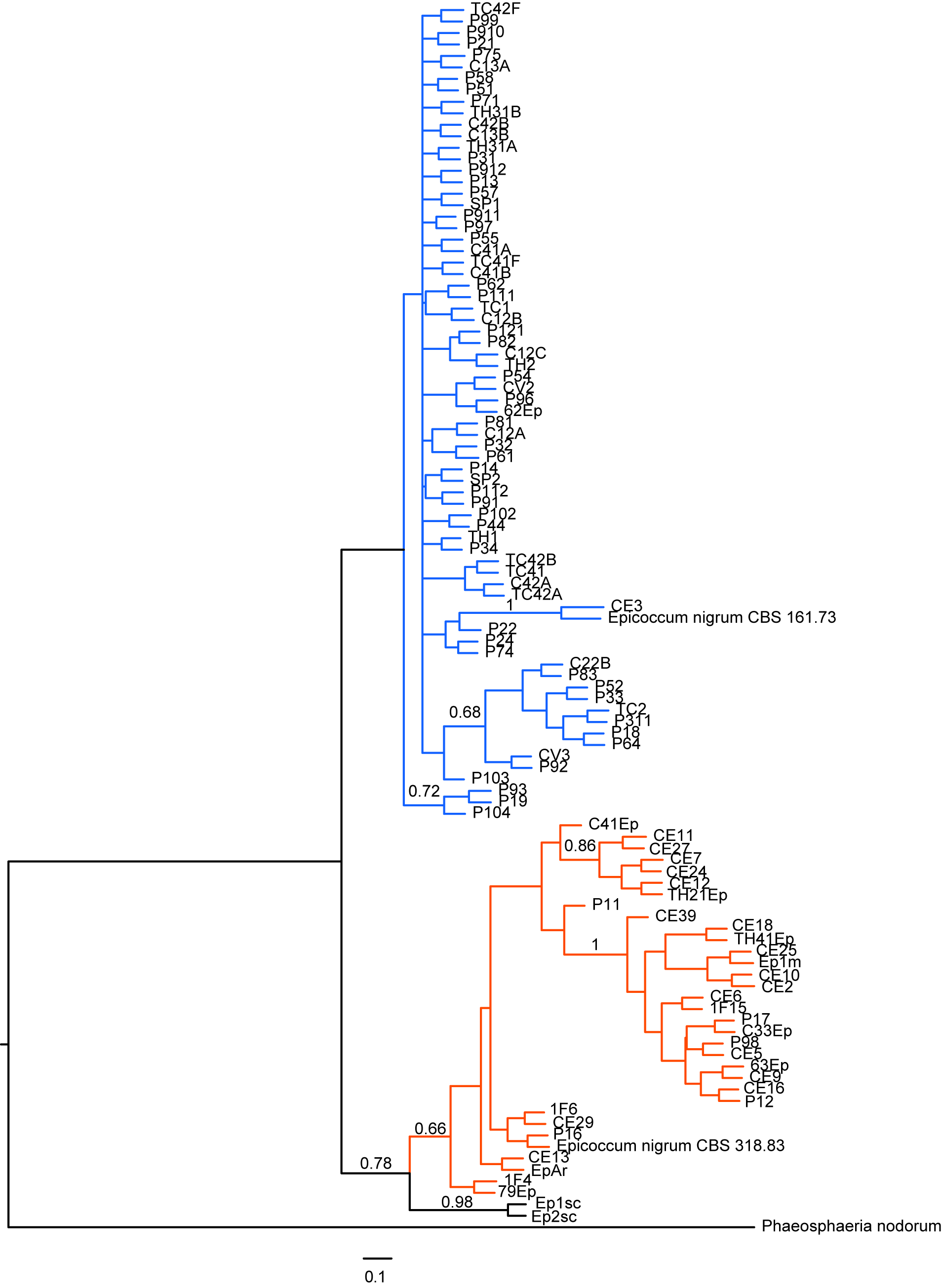

Supplement: Figure S3 — Bayesian maximum clade credibility tree inferred using {lower case beta}-tubulin partial sequences of 106 Epicoccum strains, including two E. nigrum reference strains (CBS 318.83 and CBS 161.73). P. nodorum access AY786336 was used as outgroup. The posterior probability values are shown next to relevant nodes. Orange lines represent the Epicoccum strains from group 1. Blue and black lines represent the strains from group 2. (0.57 MB TIF) [file pone.0014828.s010.tif]

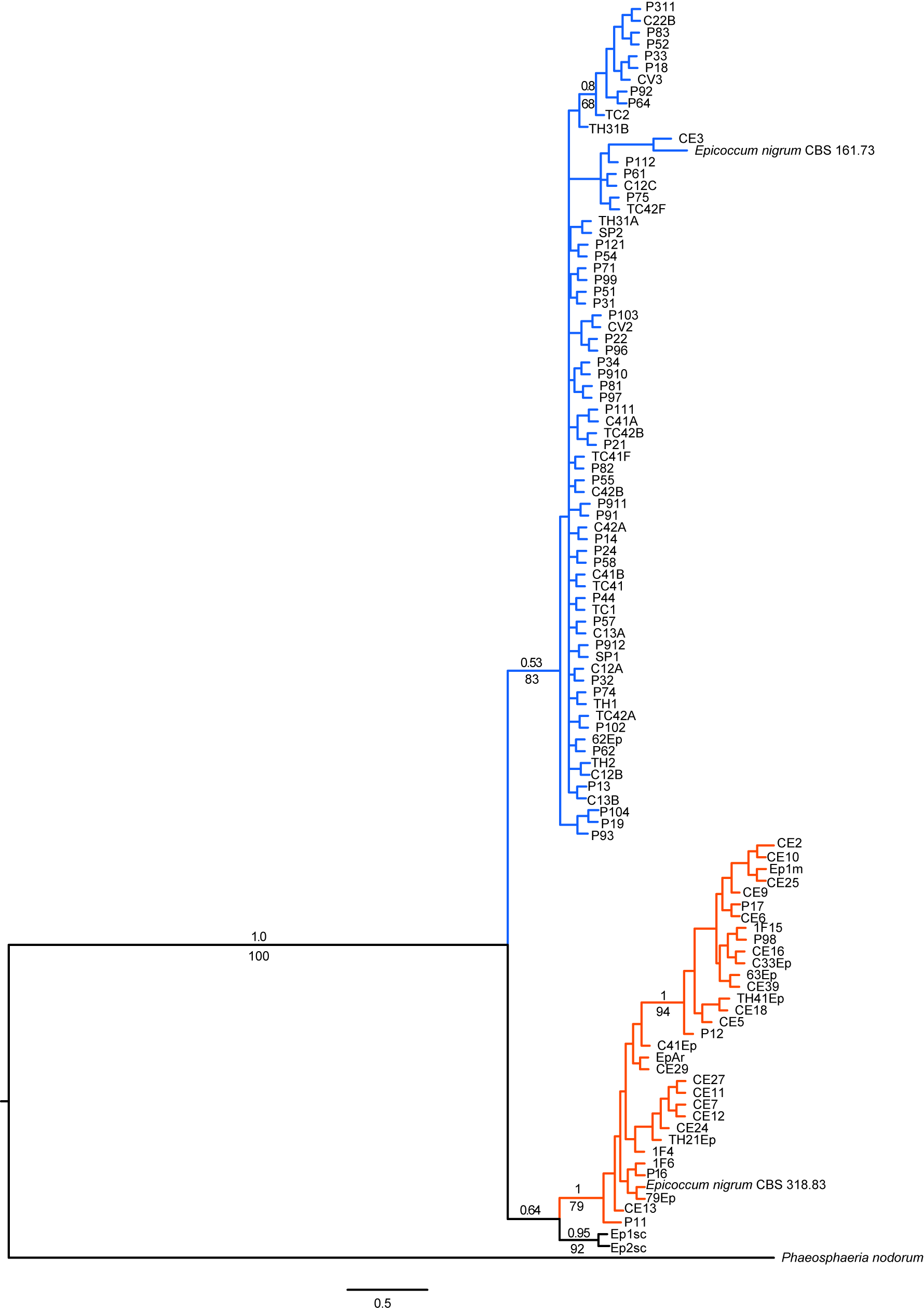

Supplement: Figure S4 — Bayesian maximum clade credibility tree inferred using concatenated ITS1-5.8S-ITS2 and β-tubulin sequences of 106 Epicoccum strains, including two E. nigrum reference strains (CBS 318.83 and CBS 161.73). P. nodorum accessions AF250830 and AY786336 were used as outgroup. The posterior probability values are shown next to relevant nodes. Orange lines represent the Epicoccum strains from group 1. Blue and black lines represent the strains from group 2. (0.50 MB TIF) [file pone.0014828.s011.tif]

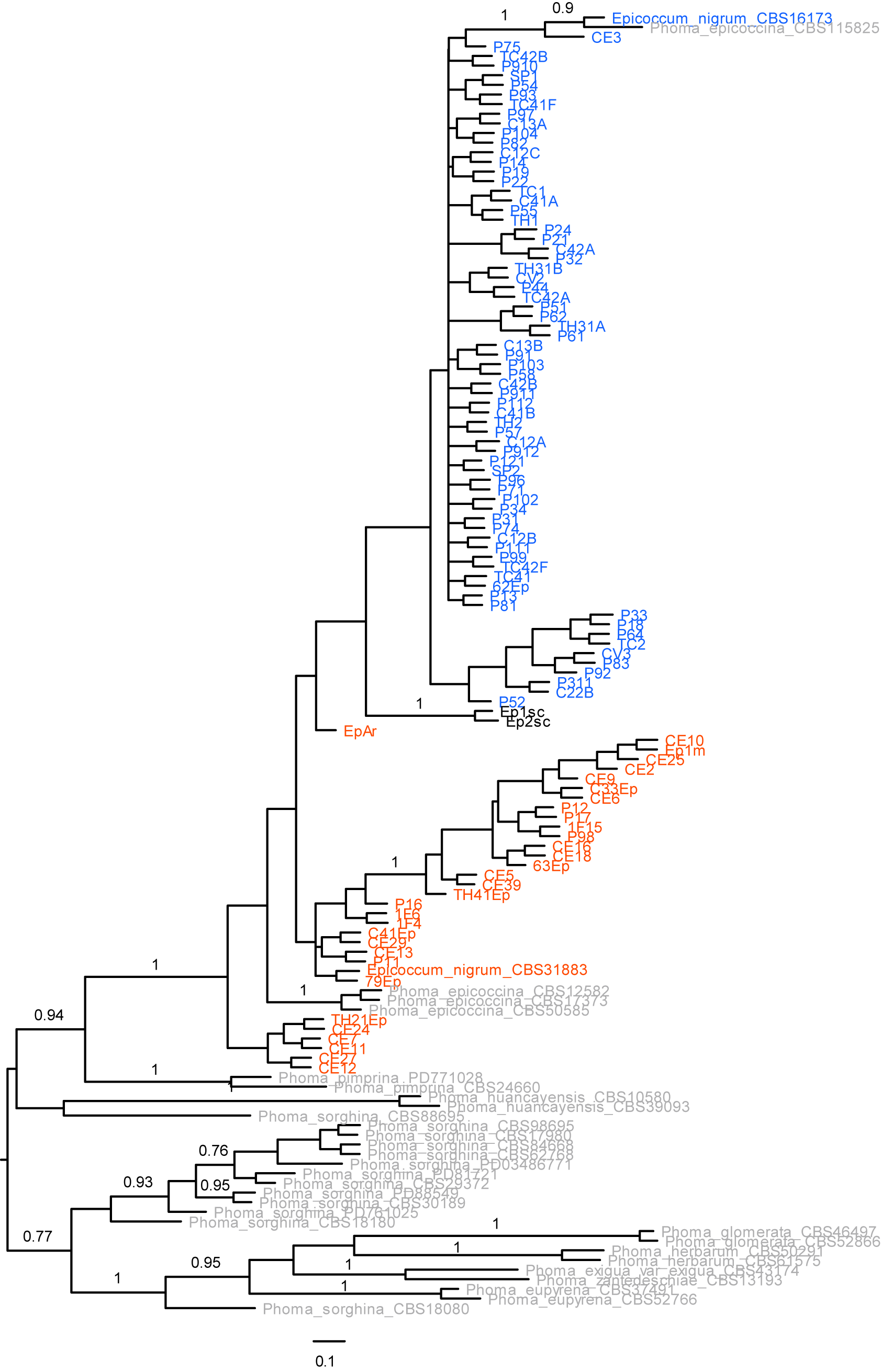

Supplement: Figure S5 — Bayesian maximum clade credibility tree inferred using β-tubulin partial sequences of 135 taxa, including 106 Epicoccum strains in the present study and other accessions of Phoma from GenBank. The β-tubulin gene sequences of P. zantedeschiae CBS 131.93 and P. exigua var. exigua CBS 431.74 were used as outgroups. The posterior probability values are shown next to relevant nodes. Strains from group 1 in this study are indicated in orange color, and strains from group 2 in this study are indicated in blue and black colors. Accessions from GenBank are typed in gray. (0.65 MB TIF) [file pone.0014828.s012.tif]

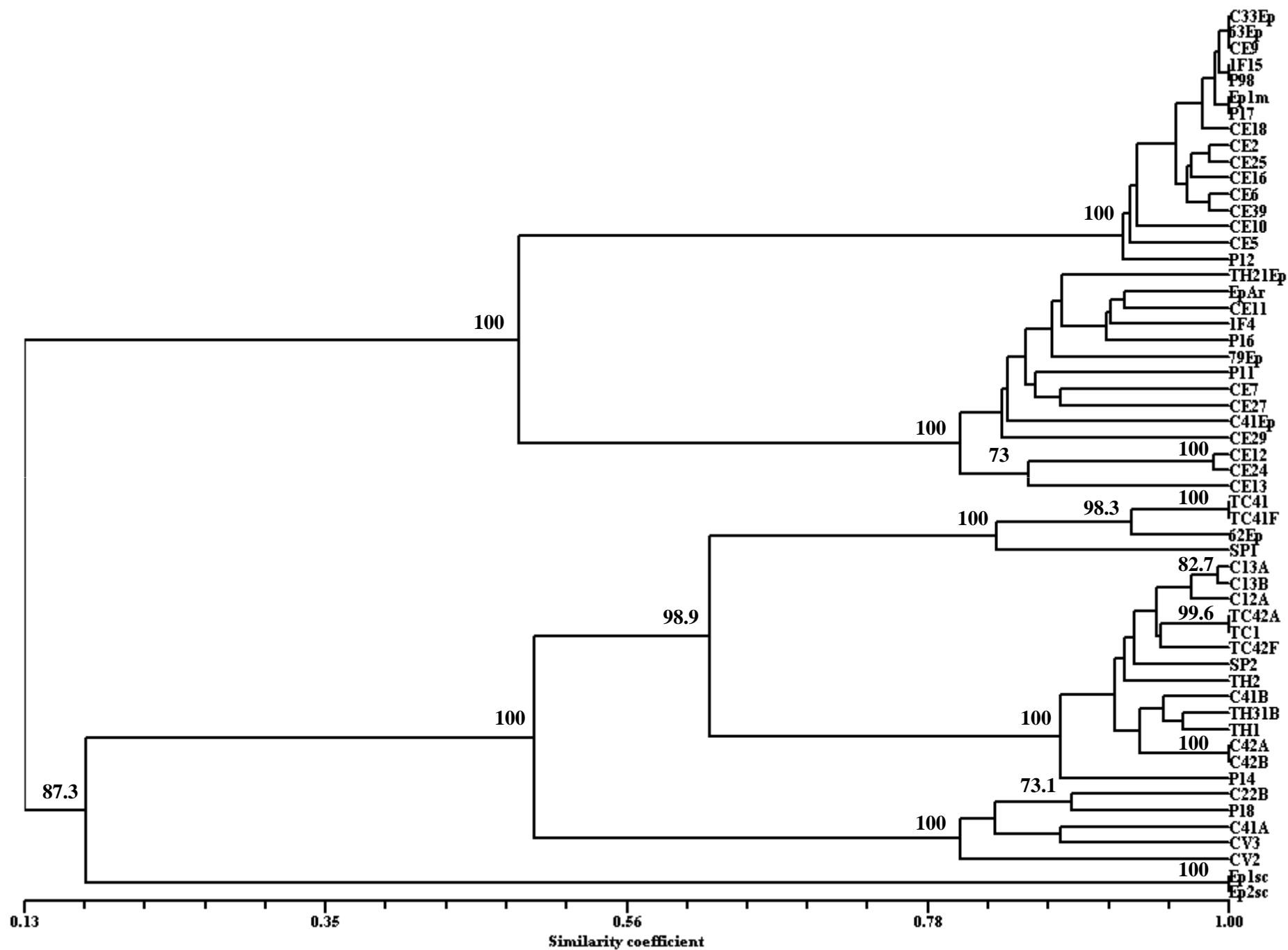

Supplement: Figure S6 — Phenogram derived from clustering analysis (UPGMA) of AFLP data of 55 Epicoccum endophytic strains based on the Dice coefficient. Bar represents the similarity coefficient. Bootstrap = 1000. (0.01 MB PDF) [file pone.0014828.s013.pdf]

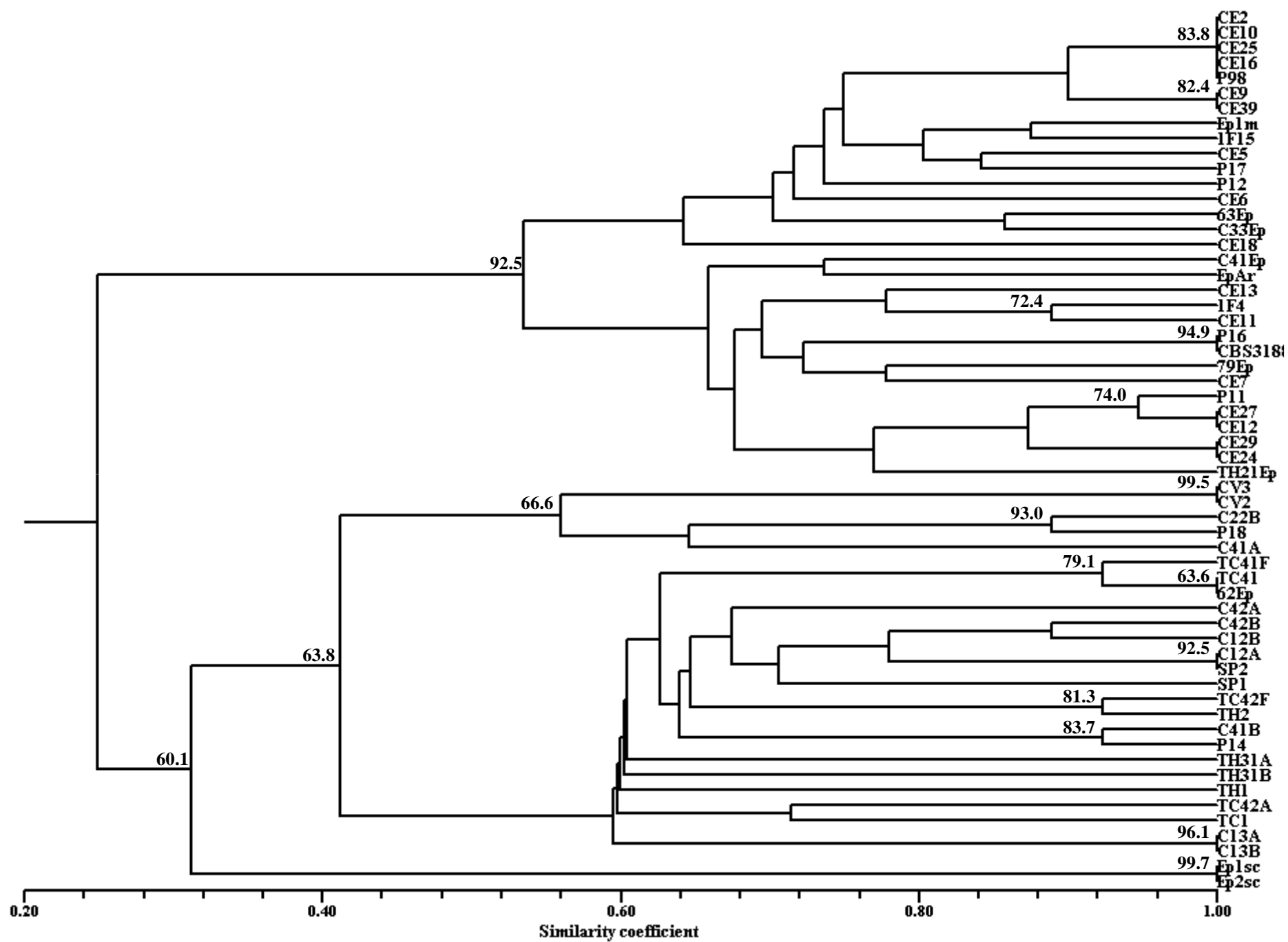

Supplement: Figure S8 — Phenogram derived from clustering analysis (UPGMA) of IGS-RFLP data of 58 Epicoccum endophytic strains based on the Dice coefficient. Bar represents the similarity coefficient. Bootstrap = 1000. (0.01 MB PDF) [file pone.0014828.s015.pdf]
